# TNAP inhibition attenuates cardiac fibrosis induced by myocardial infarction through deactivating TGF-β1/Smads and activating P53 signaling pathways

**DOI:** 10.1038/s41419-020-2243-4

**Published:** 2020-01-22

**Authors:** Lei Gao, Li-you Wang, Zhi-qiang Liu, Dan Jiang, Shi-yong Wu, Yu-qian Guo, Hong-mei Tao, Min Sun, Lin-na You, Shu Qin, Xiao-cheng Cheng, Jun-shi Xie, Guang-lei Chang, Dong-ying Zhang

**Affiliations:** Department of Cardiovascular Medicine, The First Affiliated Hospital of Chongqing Medical University, Chongqing, 400016 China

**Keywords:** Cell-cycle proteins, Myocardial infarction

## Abstract

Tissue nonspecific alkaline phosphatase (TNAP) is expressed widely in different tissues, modulating functions of metabolism and inflammation. However, the effect of TNAP on cardiac fibrosis remains controversial and needs to be further studied. The present study aims to investigate the role of TNAP on myocardial infarction (MI)-induced fibrosis and its mechanism. TNAP was upregulated in patients with MI, both in serum and injured hearts, and predicted in-hospital mortality. TNAP was also significantly upregulated after MI in rats, mostly in the border zone of the infarcted hearts combined with collagen synthesis. Administration of TNAP inhibitor, tetramisole, markedly improved cardiac function and fibrosis after MI. In the primary cultures of neonatal rat cardiac fibroblasts (CFs), TNAP inhibition significantly attenuated migration, differentiation, and expression of collagen-related genes. The TGF-β1/Smads signaling suppression, and p-AMPK and p53 upregulation were involved in the process. When p53 inhibitor was administered, the antifibrotic effect of TNAP inhibition can be blocked. This study provides a direct evidence that inhibition of TNAP might be a novel regulator in cardiac fibrosis and exert an antifibrotic effect mainly through AMPK-TGF-β1/Smads and p53 signals.

## Introduction

Our previous study reported that insulin-like peptide 6 (INSL6) inhibits cardiac fibrosis in cardiac stress models^1^. In the process, we found that tissue nonspecific alkaline phosphatase (TNAP) was upregulated both in fibrotic heart and in the kidney, and was depressed by INSL6, indicating a role on fibrosis. TNAP functions in multiple processes, including bone mineralization, vitamin B6 metabolism, neurogenesis, and inflammation^2^. Clinical trial showed that TNAP from bronchoalveolar lavage fluid can predict pulmonary interstitial fibrosis^3^. Animal experiment observed an upregulation of TNAP in fibrotic heart^4^. Latest studies of TNAP on cardiac fibrosis demonstrated controversial results^5,6^. The effects of TNAP on cardiac fibrosis and its underlying mechanism are still not clear.

Cardiac fibrosis is characterized by excessive deposition of extracellular matrix proteins in the myocardium, facilitating cardiac dysfunction^7,8^. Cardiac fibroblasts (CFs) are the main effector cells in the course^9^. CFs may proliferate, migrate, and differentiate into myofibroblasts with phenotypically expressing α-smooth muscle actin (α-SMA)^10^. Various stimuli and factors trigger the activation of signaling pathways, function on CFs, and eventually amplify the fibrotic response^7^.

Transforming growth factor (TGF)-β1/Smads is one of the key fibrogenic growth signals in cardiac fibrosis, which endows the CFs activation to acquire the ability of proliferation, migration, and collagen synthesis^10^. AMP-activated protein kinase (AMPK), the upstream of signal molecule of TGF-β1/Smads, has a protective effect during myocardial ischemia and involves in cardiac fibrosis^11,12^. A role for AMPK activation in the inhibition of the TGF-β/Smads-mediated differentiation of myofibroblasts has been described in several studies^13,14^.

Premature senescence of myofibroblasts is an essential antifibrotic mechanism and potential therapeutic target in myocardial fibrosis mediated by cell circle control^15^. AMPK can induce a cell cycle arrest by phosphorylating and stabilizing p53, a transcription factor that is activated by a plethora of stimuli including (but not limited to) DNA damage following exposure to cellular stress^16^. P53 is one of the senescence markers exerting an antifibrotic role by controlling proliferation and accumulation of CFs^15,17^. Therefore, premature senescence may be an important intervention target to improve pathological myocardial fibrosis.

In this study, we, for the first time, investigated the role of TNAP on heart injury induced by myocardial infarction (MI) both in human and in rats, and proposed a novel mechanism of how TNAP mediated cardiac fibrosis. From clinical trial in this study, we found that serum TNAP level at admission was upregulated in patients with acute MI (AMI). In another cohort, higher TNAP level at admission (TNAP ≥ 109 U/L) could predict in-hospital mortality in patients with ST-segment elevation myocardial infarction (STEMI) as an independent risk factor. TNAP also expressed in the border zone of the heart combined with collagen deposition and α-SMA expression, both in STEMI patients and MI rat hearts. Administration of TNAP inhibitor tetramisole (Tetra) improved cardiac function and alleviated cardiac remodeling in rats. TNAP inhibition also reduced collagen synthesis and CFs differentiation. Inhibition of TNAP showed heart protection effect, probably through AMPK-TGF-β1/Smad2 and p53 signaling pathways. Taken together, the present study might shed light on the role of TNAP as a therapeutic target of MI-induced cardiac fibrosis.

## Results

### Clinical trial

#### Increased TNAP in patients with MI predicted in-hospital mortality

**Fig. 1:**
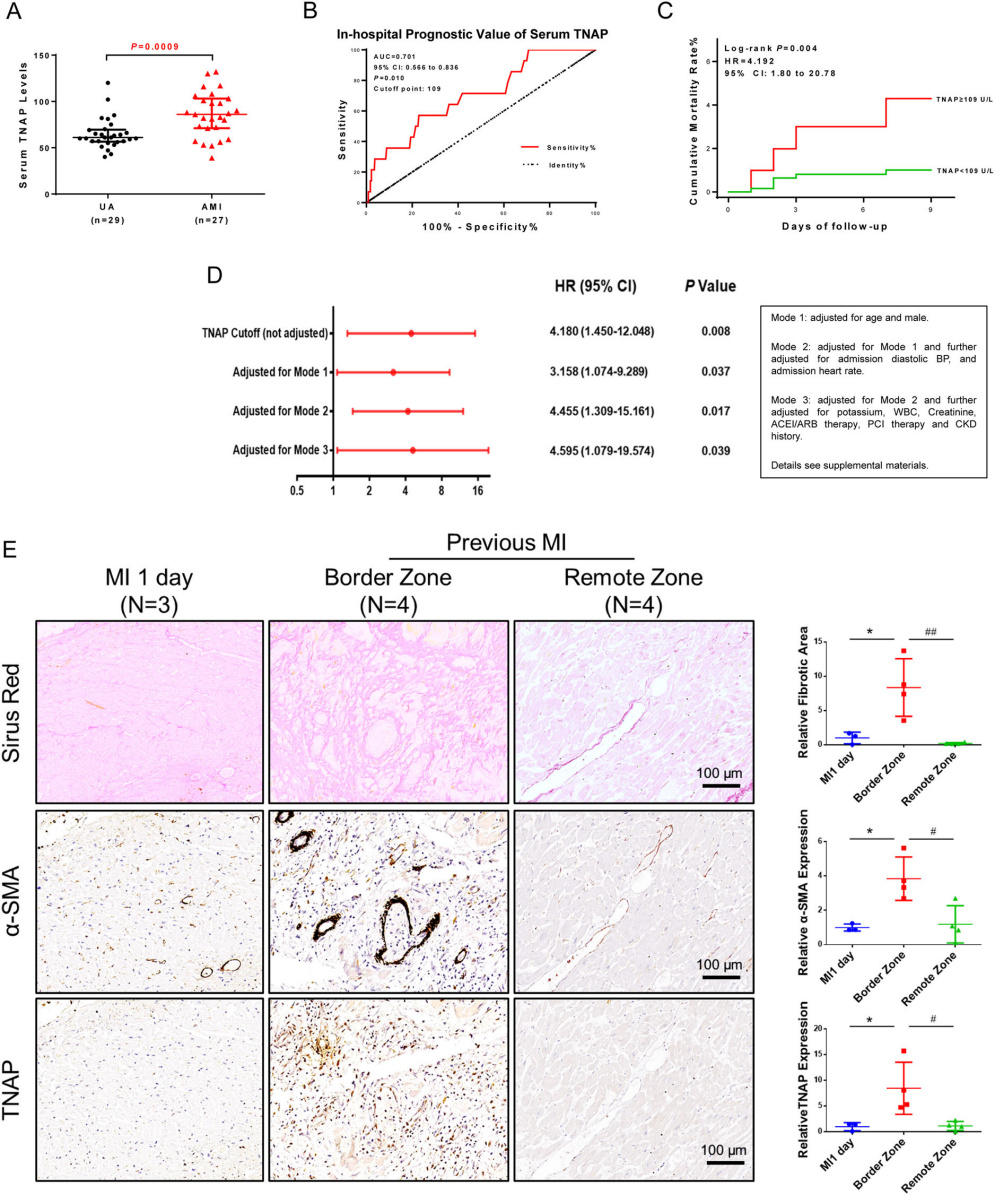
TNAP was upregulated in patients with MI both in serum and injured hearts, and predicted in-hospital mortality. **a** Serum TNAP levels in UA (*n* = 29) and AMI (*n* = 27) patients. **b** ROC curve for prediction in-hospital mortality value of serum TNAP in patients with STEMI (*n* = 826). AUC = 0.701, cutoff point = 109 U/L. **c** Cumulative in-hospital mortality rate of patients with STEMI divided by TNAP cutoff (*n* = 826). **d** Cox proportional hazards model applied to assess correlations of higher TNAP and in-hospital mortality (*n* = 826). **e** Sirus red staining and IHC staining of α-SMA and TNAP of human heart sections (*n* = 3 for MI 1 day group, *n* = 4 for previous MI group). Bar, 100 μm. *P < 0.05 vs. MI 1 day. ^#^*P* < 0.05, ^##^*P* < 0.01 vs. border zone. Abbreviations: AMI acute myocardium infarction, AUC area under curve, BP blood pressure, CKD chronic kidney disease, ROC receiver operating characteristic curve, STEMI ST-segment elevation myocardial infarction, UA unstable angina pectoris, WBC white blood cells.

TNAP upregulation leads to arterial calcification and coronary artery atherosclerosis^18^. To characterize the role of TNAP on injured heart rather than the vessel, 56 patients with acute coronary syndrome were enrolled and divided into two groups: unstable angina (UA) group (*n* = 29) and AMI group (*n* = 27). Serum TNAP level in AMI group was higher than that in UA group (Fig. 1a and Supplementary Table 1).

To assess the short-term prognostic value of TNAP, another cohort including 826 STEMI patients were registered (Supplementary Fig. 1 and Supplementary Table 2). Receiver operating characteristic curve showed that TNAP above 109 U/L probably predicted in-hospital mortality (Fig. 1b). Based on the cutoff point, the cohort was divided into two groups: TNAP high group (≥109 U/L) and low group (<109 U/L). Patients in TNAP high group had a significantly higher in-hospital mortality than the patients in the low group (hazard ratio = 4.192, *P* = 0.004) (Fig. 1c). Three modes of Cox proportional hazards regression adjusted factors including age, sex, admission diastolic blood pressure, heart rate, potassium, white blood cell, creatinine, angiotensin-converting enzyme inhibitors (ACEI/ARB) therapy, percutaneous transluminal coronary intervention therapy, and chronic kidney disease history. All modes analyses clarified that increased serum TNAP level may be an independent risk factor of in-hospital mortality in patients with STEMI (Fig. 1d and Supplementary Tables 3 and 4).

To assess whether TNAP is elevated in the fibrotic remodeling process after heart injury, serial heart sections of MI patients were used to verify the relationship between TNAP expression and cardiac fibrotic deposition, and myofibroblasts marker, α-SMA. Immunohistochemical (IHC) results showed that TNAP and α-SMA were less expressed in the heart of patients after 1 day of MI, whereas both TNAP and α-SMA were upregulated in the border zone of the heart combined with collagen deposition in patients with previous MI (Fig. 1e). These results suggested that TNAP was elevated when patients suffered with MI and may further play a role on cardiac fibrosis process.

In the following in vitro and in vivo experiments, we further investigated the effects and underlying mechanisms of TNAP on cardiac fibrosis in MI model.

### Animal study

#### Heart TNAP upregulated and mainly expressed in the border zone of post-MI hearts combined with collagen deposition in rats

**Fig. 2:**
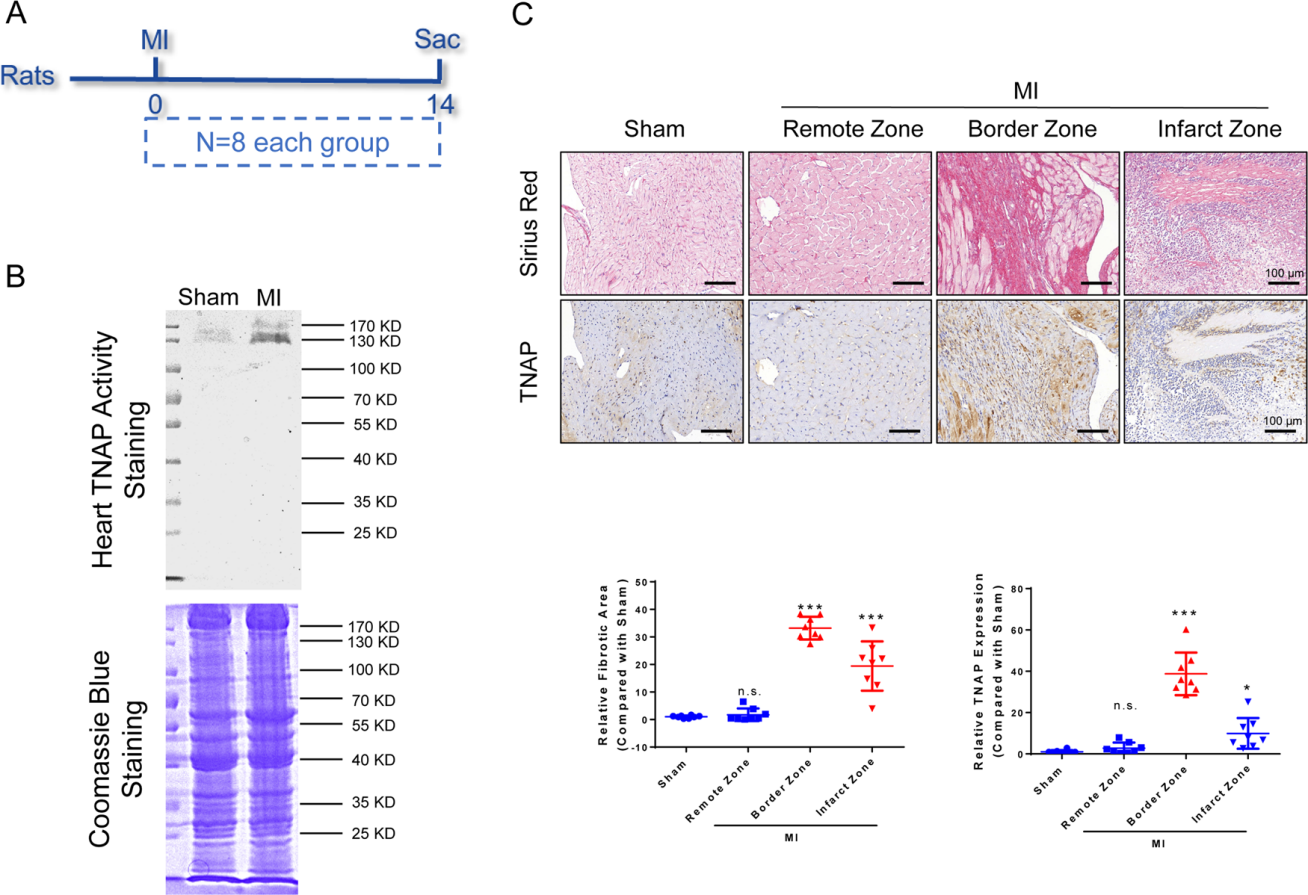
TNAP was mainly expressed in the border zone of post-MI hearts combined with collagen deposition in SD rats. **a** Time axis of SD rats. Rats were performed MI or sham operation at 0-day time point and sacrificed at 14-day time point. **b** In-gel heart TNAP activity assessed by BCIP/NBT method. TNAP around 130 KD (*n* = 8, each). **c** Sirius red staining and IHC staining of TNAP were performed in serial left ventricle sections. Bar 100 μm (*n* = 8, each). Relative fibrotic area and relative TNAP expression were used to analyze the differences among groups. NS, *P* > 0.05, *P < 0.05, ***P < 0.001 vs. Sham.

To examine the expression, activity, and location of TNAP, we used the hearts of Sprague–Dawley (SD) rats undergoing MI surgery after 1, 3, 7, 14, and 28 days (Supplementary Fig. 2a). The heart TNAP activity and expression were elevated in a time-dependent manner post MI, which was combined with collagen synthesis and α-SMA expression (Supplementary Fig. 2b, c, e, f). However, serum TNAP activity among groups had no significant difference (Supplementary Fig. 2d). We chose the time point of 14 days after MI to estimate the heart TNAP activity and expression. TNAP activity was upregulated compared with sham group (Fig. 2b) and the expression was highly assembled in the border zone of post-MI heart combined with collagen deposition in the same parts (Fig. 2c).

#### TNAP inhibition improved cardiac function and attenuated pathological remodeling post MI in rats

Experiments were performed to test the effect of TNAP inhibitor, Tetra. Rats were sacrificed at the same time point after Tetra administration (intraperitoneal (i.p.) injection, 11 mg/kg/day, once a day) at 0, 7, 14, and 28 days (Supplementary Fig. 3a). TNAP activities were assessed by two methods. Tetra administration continuously inhibited heart TNAP activity during the whole 28 days (Supplementary Fig. 3c, d), while temporarily inhibited serum TNAP activity in 14 days (Supplementary Fig. 3b). Collectively, TNAP activity could be validly inhibited before operation after Tetra dosing for 7 days.

**Fig. 3:**
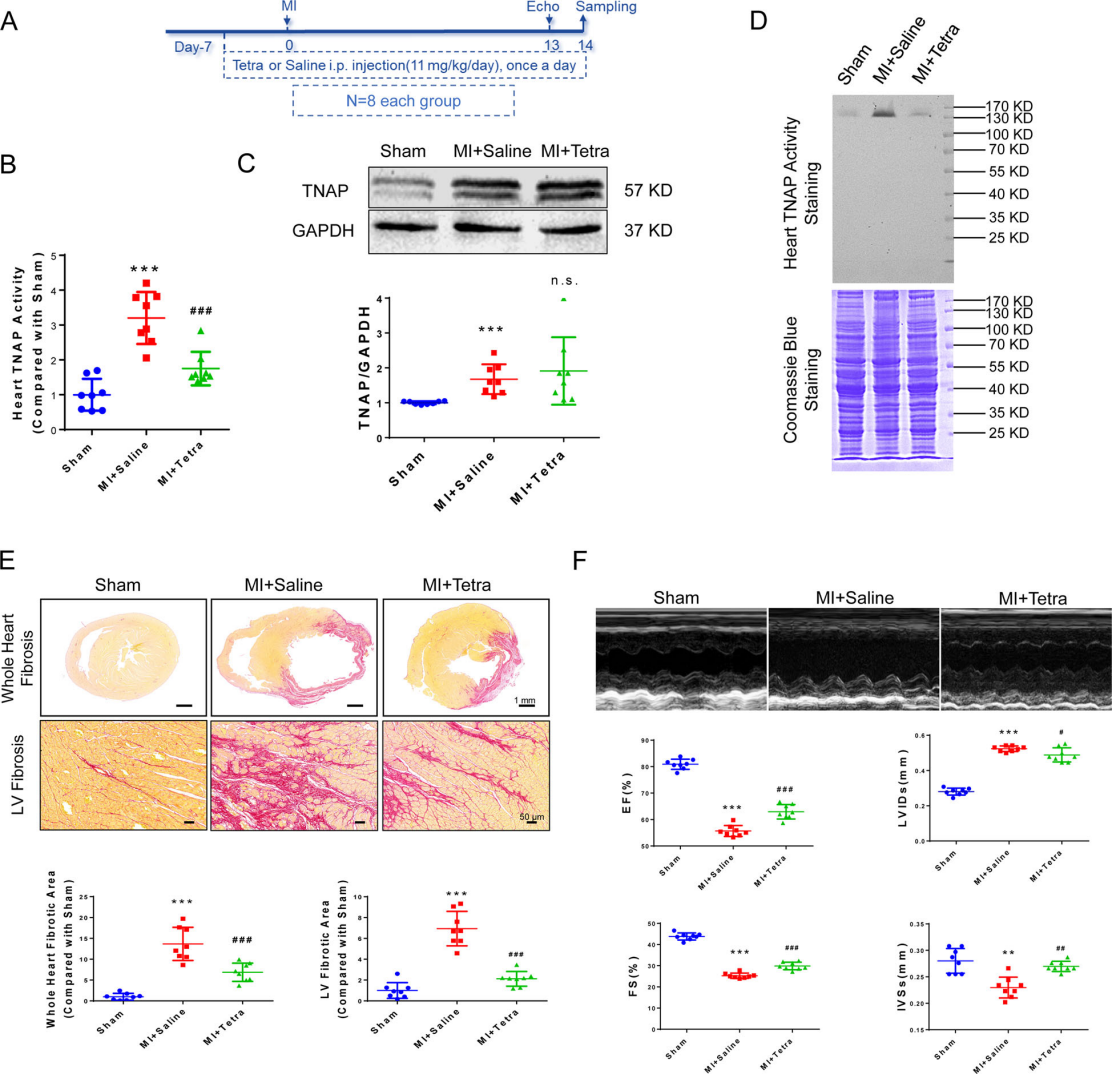
TNAP inhibition improved cardiac function and attenuated pathological remodeling post MI in SD rats. **a** Time axis of SD rats. Tetra or saline was administrated to rats from 7 days before operation till sampling (14 days after operation). Echocardiography was performed 13 days after operation. **b** Heart TNAP activity was assessed by alkaline phosphatase assay kit. **c** Western blotting was performed to analyze heart TNAP expression. The inactivated TNAP bands were around 57 KD. Statistical result using relative gray values (compared with sham) of the blotting was below the band. **d** In-gel TNAP activity was assessed by BCIP/NBT method. The activated TNAP was around 130 KD. **e** Sirus red staining of whole heart fibrosis (bar 1 mm) and LV fibrosis (bar 50 μm). Statistical analyses of fibrotic area were shown below (*n* = 8, each). **e** Doppler echocardiography was used to assess hemodynamic and anatomic changes among sham, MI + Saline, and MI + Tetra groups. ***P < 0.001 vs. Sham. ^###^ *P* < 0.001 vs. MI + Saline (*n* = 8, each). Abbreviations: EF ejection fraction, FS fractional shortening, IVS interventricular septal thickness at end systole, LVID left ventricular end systolic internal diameter.

Tetra was administrated to rats from 7 days before operation till sampling (14 days after operation) (Fig. 3a). Heart TNAP activity was validly inhibited in MI + Tetra group (Fig. 3b, d), whereas TNAP protein level was not affected (Fig. 3c). Myocardial infarcted and fibrotic area was significantly reduced in MI + Tetra group compared with MI + Saline group (Fig. 3e). Left ventricular (LV) anterior wall impulse and heart function parameters of ejection fraction (EF), fractional shortening (FS), LV end systolic internal diameter (LVIDs), and interventricular septal thickness at end systole (IVSs) (Fig. 3f) were improved in MI + Tetra group.

#### TNAP inhibition reduced fibrotic-related protein expression in post-MI hearts in rats

**Fig. 4:**
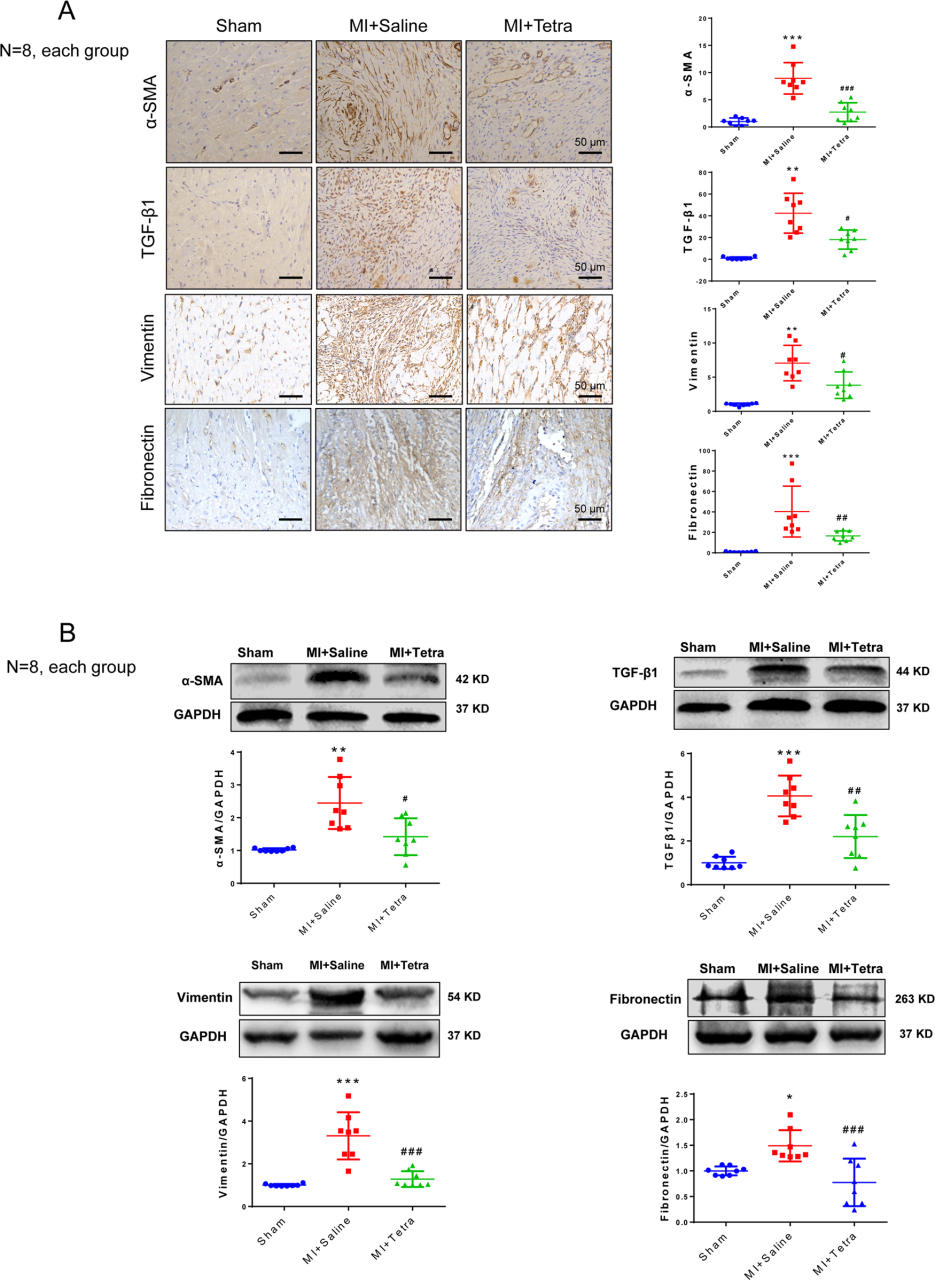
TNAP inhibition reduced fibrotic-related protein expression in post-MI hearts in rats. **a** Left panel: IHC staining of α-SMA, TGF-β1, Vimentin, and Fibronectin. Bar 50 μm. Right panel: statistical results of IHC by assessing mean optical density of the brown area (*n* = 8, each). **b** Western blotting of α-SMA, TGF-β1, Vimentin, and Fibronectin. Statistical results using relative gray values of the blotting were below the band (*n* = 8, each). *P < 0.05, **P < 0.01, ***P < 0.001 vs. Sham. ^#^*P* < 0.05, ^##^*P* < 0.01, ^###^ *P* < 0.001 vs. MI + Saline.

We focused the pathological change post MI on fibrotic-related proteins including TGF-β1, which stimulates α-SMA and Vimentin expression leading to progression of cardiac fibrosis^7^. IHC and western blotting were performed. TGF-β1, α-SMA, Vimentin, and fibronectin were upregulated in MI + Saline group compared with those in Sham group. Inhibiting TNAP significantly suppressed TGF-β1, α-SMA, Vimentin, and fibronectin expression (Fig. 4). Inhibiting TNAP also upregulated p-AMPK and deactivated Smads signaling by dephosphorylation of Smad2 and blocking Smad3 nuclear import (Supplementary Fig. 4b, c).

### In vitro study

#### TNAP inhibition attenuated TGF-β1-induced CFs differentiation and collagen synthesis

**Fig. 5:**
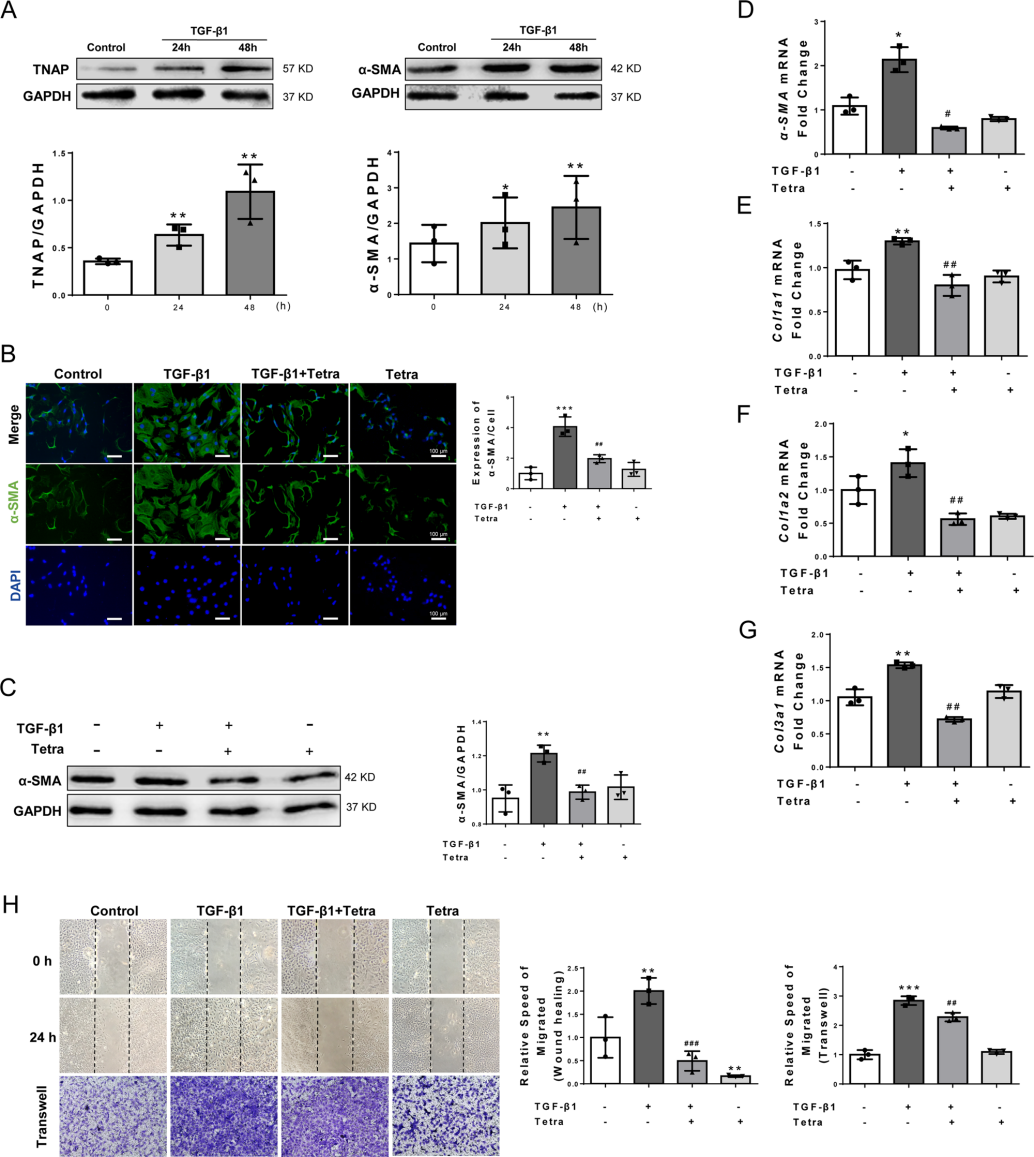
TNAP inhibition attenuated TGF-β1-induced differentiation and collagen synthesis in vitro. **a** TGF-β1 induced TNAP and α-SMA expression after incubation for 24 h and 48 h (*n* = 3). Statistical results using relative gray values of the blotting were below the band. **b** Immunofluorescence staining of α-SMA after TGF-β1 and/or Tetra treatment for 24 h. Green stands for α-SMA and blue stands for DAPI. Bar, 100 μm. Quantification of α-SMA expression per cell were analyzed (*n* = 3). **c** Western blotting of α-SMA after 24 h treatment of TGF-β1 and/or Tetra (*n* = 3). Statistical result was in the right panel. **d**–**g**. RT-PCR results after incubation for 24 h (**d**) and 48 h (**e**, **f**, **g**) (*n* = 3). **h** Migration of CFs measured by wound-healing assay and Transwell assay. For wound-healing assay, cells were cultivated in serum-free medium. Both the assays were assessed after incubation for 24 h and relative speed of migration were measured (*n* = 3). *P < 0.05, **P < 0.01, ***P < 0.001 vs. Control group. ^#^*P* < 0.05, ^##^*P* < 0.01, ^###^*P* < 0.001 vs. TGF-β1 group.

To determine whether TGF-β1 can upregulate the expression of TNAP and α-SMA, neonatal rat CFs were incubated with 10 ng/ml TGF-β1 for 24 h and 48 h. Both TNAP and α-SMA were significantly upregulated by TGF-β1 in a time-dependent manner (Fig. 5a). We further investigated the effect of TNAP on CFs differentiation induced by TGF-β1. CFs were pretreated with 1 mM TNAP inhibitor Tetra for 30 min and then treated with 10 ng/ml TGF-β1 for 24 and 48 h, respectively. Protein and mRNA levels of α-SMA were used to evaluate the differentiation of CFs. Tetra pretreating significantly reduced TGF-β1-induced α-SMA expression, suggesting the differentiation of CFs was attenuated by TNAP inhibition (Fig. 5c, d). This effect was further verified by immunofluorescence (Fig. 5b).

*Col1a1*, *Col1a2*, and *Col3a1* mRNA quantification assays were used to evaluate the collagen synthesis ability of CFs. Results showed that TGF-β1 enhanced *Col1a1*, *Col1a2*, and *Col3a1* mRNA expression, whereas this effect was abolished when CFs were pre-incubated with Tetra (Fig.5e–g).

Migration of CFs was measured by transwell and wound-healing assays. Results showed that Tetra pre-incubation significantly inhibited TGF-β1-induced CFs migration (Fig. 5h). All these results suggested that TNAP inhibition directly ameliorated TGF-β1-induced myofibroblast differentiation, collagen synthesis, and cell migration.

#### Activation of AMPK and deactivation of TGF-β1/Smad2 were involved in TNAP inhibition, P53/cyclinE1 might be a potential target pathway

**Fig. 6:**
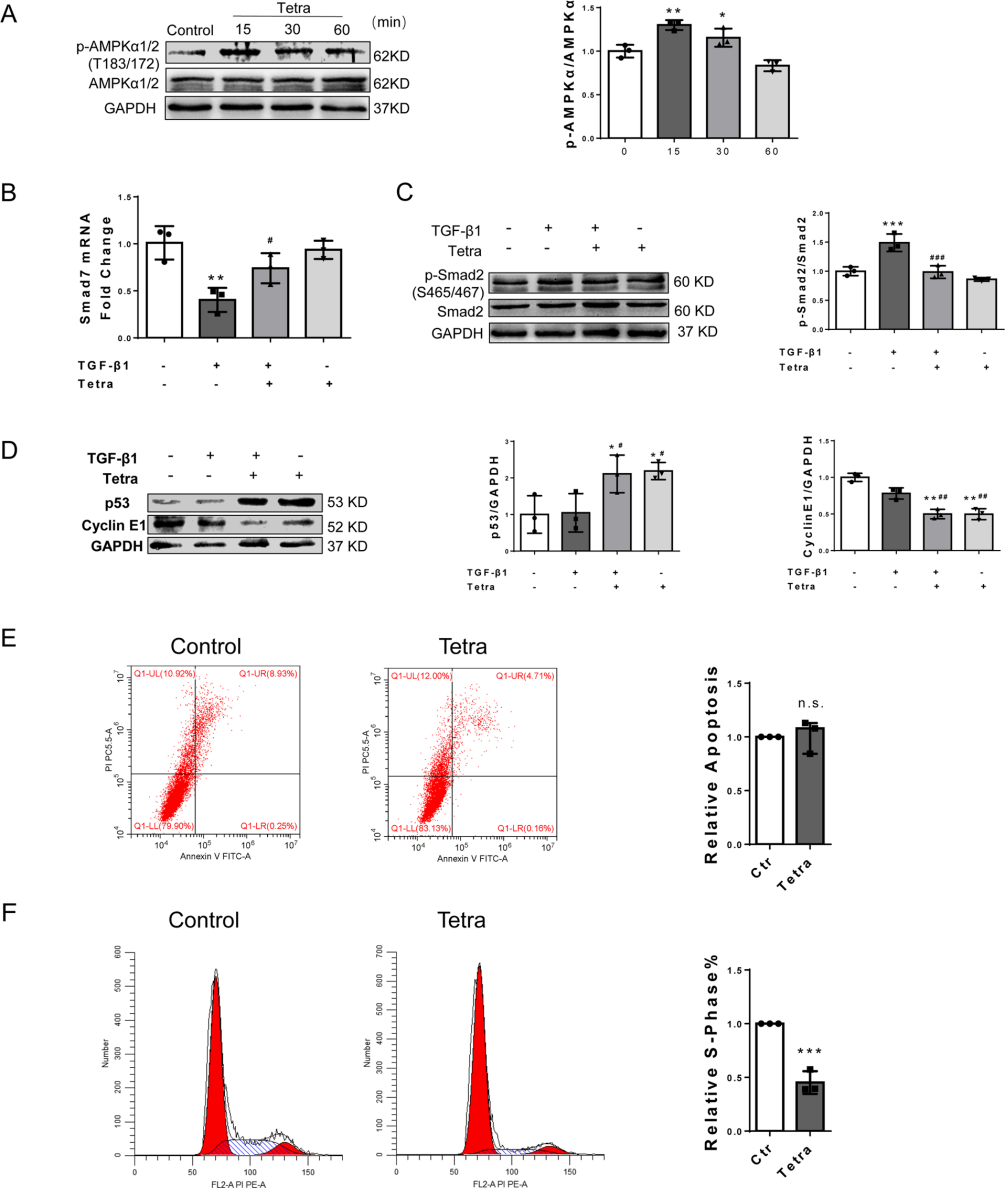
Activation of AMPK and AKT, deactivation of TGF/Smads, and activation of p53 were involved in TNAP inhibition. **a** p-AMPK, AMPK, and GAPDH expression after Tetra incubation for 15, 30, and 60 min (*n* = 3). Statistical result was in the right panel. **b** *Smad7* mRNA expression after TGF-β1 incubation for 72 h (*n* = 3). **c** p-Smad2, Smad2, and GAPDH expression after TGF-β1 incubation for 1 h (*n* = 3). Statistical result was in the right panel. **d** p53 and CyclinE1 expression after TGF-β1 incubation for 24 h (*n* = 3). Statistical results were in the right panel. **e** Apoptosis analyzed by flow cytometry after Tetra treatment for 24 h. Total proportion of apoptotic cell was analyzed and shown in the right panel (*n* = 3). **f** Cell cycle assessed by flow cytometry after Tetra treatment for 24 h. The percentages of S-phase cells were analyzed and shown in the right panel (*n* = 3). *P < 0.05, **P < 0.01, ***P < 0.001 vs. control group. ^#^*P* < 0.05, ^##^*P* < 0.01, ^###^*P* < 0.001 vs. TGF-β1 group.

AMPK signaling plays an important role in cardiac fibrosis regulation and myofibroblast differentiation. To determine whether TNAP inhibition can activate AMPK, CFs were incubated with 1 mM Tetra for 15, 30, and 60 min, respectively. Phosphorylation of AMPKα1/2 (Thr183/172) was significantly increased in Tetra-treated CFs at 15 and 30 min (Fig. 6a). These results were in accord with our in vivo study found (Supplementary Fig. 4a).

Activating AMPK may regulate TGF-β1/Smad2 signaling through activating Smad7^13,19^. CFs were pretreated with 1 mM Tetra for 30 min and then incubated with 10 ng/ml TGF-β1 for 1 h, 72 h to detect the Smad2 phosphorylation and *Smad7* mRNA expression, respectively. Results showed that Smad2 phosphorylation (Ser465/467) was significantly enhanced by TGF-β1. Pre-treatment with Tetra markedly diminished this effect of TGF-β1 (Fig. 6c). Correspondingly, Smad7, a dephosphorylate factor of Smad2, was downregulated by TGF-β1 at the transcriptional level. Inhibiting TNAP significantly upregulated *Smad7* mRNA expression level (Fig. 6b).

Premature cellular senescence plays a vital role in tissue remodeling, including cardiac fibrosis^15^. We investigated the biomarkers of cell senescence, p53 and its downstream molecule cyclinE1, to show whether cell premature occurred in TNAP inhibition of CFs. We did not find significant change of p53 and cyclinE1 after TGF-β1 stimulation. However, p53 was upregulated and cyclinE1 was downregulated after Tetra pre-incubation with and without TGF-β1 (Fig. 6d). These results suggested that p53 signaling might be a potential target that mediated antifibrotic effect of TNAP inhibition in CFs through a TGF-β1/Smads-independent way.

P53-mediated senescence could be the antifibrotic mechanism by arresting cell cycle but not apoptosis^20,21^. To show this process, we performed flow cytometry to examine the cell cycle and apoptosis after TNAP inhibition. Results showed that inhibition of TNAP could inhibit CFs cell cycle but not apoptosis (Fig. 6e, f).

#### Inhibition of TNAP mitigated hypoxia-induced fibrotic changes in CFs, probably through p53 signaling pathway

**Fig. 7:**
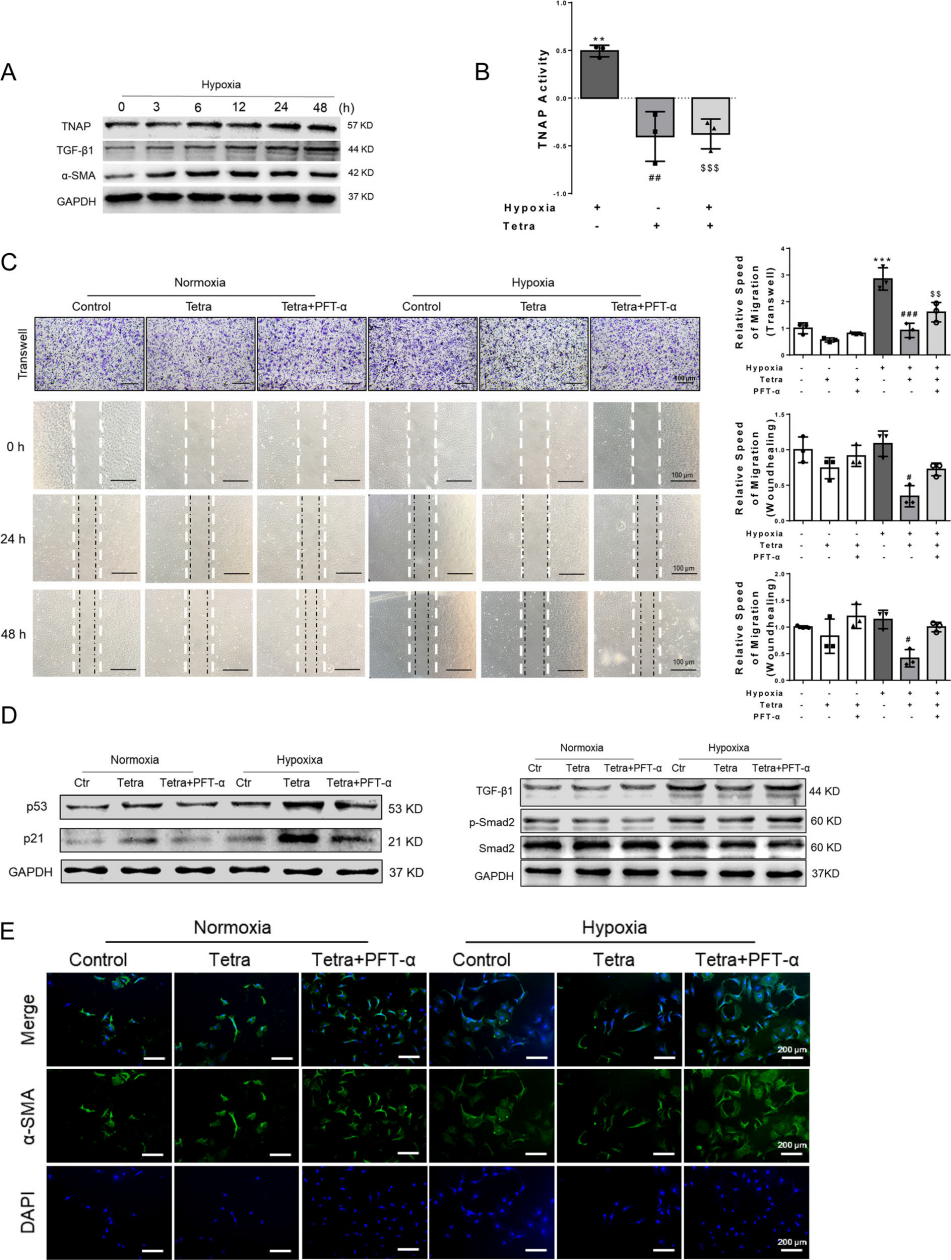
Inhibition of TNAP mitigated hypoxia-induced fibrotic changes in CFs, probably through p53 signaling pathway. **a** TNAP, TGF-β1, α-SMA, and GAPDH expression after hypoxia (1% O_2_) (*n* = 3, each). **b** TNAP activity assessed by alkaline phosphatase assay kit after hypoxia for 24 h (*n* = 3, each). **c** Migration of CFs measured by Transwell assay after 24 h and wound-healing assay after 24 h and 48 h. Statistical results of relative migration speed were analyzed (*n* = 3, each). Bar, 100 μm. For wound-healing assay, cells were cultivated in serum-free medium. **d** p53, p21, TGF-β1, p-Smad2, Smad2, and GAPDH expression measured by western blotting after hypoxia for 24 h (*n* = 3, each). **e** α-SMA expression measured by immunofluorescence. (*n* = 3, each). Green stands for α-SMA and blue stands for DAPI. Bar, 200 μm. **P < 0.01, ***P < 0.001 vs. control group. ^#^*P* < 0.05, ^##^*P* < 0.01, ^###^*P* < 0.001 *vs*. hypoxia group. ^$$^*P* < 0.01, ^$$$^*P* < 0.001 vs. hypoxia + Tetra group.

To inquire the independent role of p53, hypoxia cultural CFs was used to mimic the pathological process of MI in vitro. During hypoxia (1% O_2_) incubation, TNAP, TGF-β1, and α-SMA were upregulated in a time-dependent manner (Fig. 7a). TNAP activity was also increased after hypoxia for 24 h and Tetra significantly blocked this process (Fig. 7b). The cellular morphology was also changed by hypoxia, whereas Tetra incubation well-protected this process (Supplementary Fig. 5).

Wound-healing and transwell assay were used to explore the migration ability of CFs in the context of hypoxia. Results showed that Tetra incubation alleviated hypoxia-induced CFs migration, whereas p53 inhibitor (PFT-α) well-diminished this effect (Fig. 7c) measured by transwell after incubation for 24 h. This phenomenon was also observed in wound-healing assay after hypoxia cultivation for 24 h and 48 h that Tetra incubation well -blocked the migration induced by hypoxia. P53-inhibited CFs had a tendency to migrate more compared with the Tetra group but no statistical significance was found.

PFT-α could suppress p53 transcription stated in the manufacturer’s instruction and certified by several researches^22,23^. We found that Tetra incubation upregulated the expression of p53 and p21, both in normoxia and hypoxia with more pronounced increase in hypoxic environments. TGF-β1 and p-Smad2 were upregulated by hypoxia, whereas TNAP inhibition mitigated the effect of hypoxia on TGF-β1 and Smad2. In addition, p53 inhibitor PFT-α well-diminished the protection effect of TNAP inhibition (Fig. 7d).

Expression of α-SMA was examined by immunofluorescence that Tetra incubation inhibited hypoxia-induced α-SMA expression, while PFT-α diminished this effect (Fig. 7e).

As PFT-α has a p53-independent cytoprotective effect^24^, key experimental results were repeated and showed in Supplementary Fig. 6 using genetic knockdown of P53 by small interfering RNA (siRNA). Si-P53 significantly downregulated p53 with the presence or absence of Tetra. The downstream molecule p21 was also downregulated (Supplementary Fig. 6a, b). Migration ability of CFs were measured by transwell and wound-healing assays after incubation for 24 h. Hypoxia-induced migration was decelerated after Tetra treating, which was diminished by si-P53 (Supplementary Fig. 6c). Collectively, inhibition of TNAP exerted antifibrotic role probably through p53 signaling pathway.

## Discussion

TNAP has long been recognized as a calcifying marker coded by *ALPL* gene^25^, but our results from clinical research suggested TNAP may be involved in fibrotic remodeling post MI. First, we found that TNAP was upregulated in patients with AMI compared with patients with UA (Fig. 1a) and it was also an independent risk factor for in-hospital death of STEMI patients (Fig. 1b–d), which was in accordance with previous report^26^. We further examined TNAP in donated heart sections and found the assembling of TNAP was mainly in the border zone of MI hearts along with the collagen deposition and α-SMA expression. The results were confirmed by MI model of SD rats (Fig. 2 and Supplementary Fig. 2), which was consistent with Martin’s reports in failing hamster hearts and ischemic porcine hearts^4^. It verified our assumption that TNAP may play a role in cardiac fibrosis.

TNAP upregulation was observed starting at day 3, peaked at day 14, and decreased at day 28 in our MI animal model (IHC results in Supplementary Fig. 2c). Reports showed that the early inflammatory phase after MI (~4 days in mice) is characterized by a robust innate and adaptive immune cell infiltration and tissue digestion. This is subsequently followed a phase of resolution, myofibroblast proliferation, and wound repair (lasting ~10–14 days in mice)^27^. We found a consistent time frame change in TNAP and cardiac fibrosis, both peak at day 14. The further study to investigate the inflammatory or immune factors stimulating TNAP is in progress.

Our results clarified that TNAP inhibitor, Tetra (11 mg/kg/day), could suppress serum and heart TNAP activity after 7 days i.p. injection in SD rats (Supplementary Fig. 3). Whole heart fibrotic level and tissue TNAP activity of post-MI rats were suppressed by Tetra administration, combined with the improvement of the echocardiography parameters including EF, FS, LVIDs, and IVSs (Fig. 3).

TGF-β1 signaling is crucial for the induction and maintenance of activated fibroblast phenotype through the Smad2/3 pathways^7,28^. In the present study, we found TNAP inhibition significantly suppressed the myofibroblast differentiation and collagen deposition both in post-MI hearts and in TGF-β1-treated CFs evidenced by the reducing expression of α-SMA, vimentin, collagen-related genes, and migration. Inactivating TGF-β1/Smads signaling might be involved in TNAP inhibition according to the in vitro study results (Figs. 5 and 6). We further tested whether Smads signaling was activated in context of MI injury. As expected, TNAP inhibition inactivated Smad2 and blocked Smad3 transportation to the nucleus in MI-induced rat heart (Supplementary Fig. 4). Reduction TNAP by short hairpin RNA was reported significantly reduced prostate cancer cells migration with lower vimentin expression^29^. Rodionov et al.^6^ also reported in a mouse model of coronary artery disease that endothelial TNAP overexpression transgenic mice associated with increased myocardial fibrosis. Both were consistent with our results. However, by using *TNAP*^cre^*Tgfβr2*^fl/fl^ mice and C2C12 myoblasts, Arno and Galli^5^ claimed that the TNAP limited TGF-β-dependent cardiac fibrosis by inactivating Smad2/3. The article reported that deletion of *Tgfβr2* in TNAP-positive cells exerted antifibrotic role after AngII stimulation. It only proved that *Tgfβr2* was indispensable in AngII-induced fibrosis without solid evidence of TNAP suppressing cardiac fibrosis.

Multiple types of TNAP-positive cells in the heart express *Tgfβr2* with different function after heart injury^9^. CFs, cardiomyocytes, and endothelial cells contribute to cardiac fibrosis after injury^30^, whereas bone marrow-derived suppressor cells (MDSCs) exerts antifibrotic role by suppressing inflammation^31,32^. TNAP function might be a diversity in these cells and its cardiac fibrosis contribution needs to be discriminated in conditional knockout/in TNAP mice. In the report by Arno and Galli^5^, C2C12 cells with no detectable TNAP were used to identify the relationship between TNAP and Smad2/3, but it is not the typical fibrotic-mediated cells in the heart. Studies of TNAP gene overexpression and knockdown mice were proceeded systematically (data not show).

AMPK signaling is pivotal in fibrotic regulation. We found that inhibition of TNAP promoted AMPKα1/2 phosphorylation at Thr183/172 in CFs (Fig. 6). The possible mechanism underlying AMPK activation may attribute to the AMP-hydrolyzing effect of TNAP confirmed by studies in different type of cells and the effect can be eliminated by TNAP pharmacological inhibitor MLS-0038949 and levamisole^33–36^. Gowans et al.^37^ reported AMP is a true physiological regulator of AMPK by both allosteric activation and enhancing net phosphorylation. Due to the AMP-hydrolyzing effect of TNAP, the inhibition of TNAP activated AMPK signaling in CFs, probably mediated by AMP concentration change.

The activated AMPK signaling is also a critical regulator of TGF-Smad signaling by activating Smad7, which resulted in dephosphorylation of Smad2/3^13,19^. Our results further support this point that inhibition of TNAP upregulated expression of *Smad7* mRNA level while suppressing the phosphorylation of Smad2. We supposed the TNAP-AMP-AMPK-TGF-β/Smads signal may contribute to the cardiac fibrosis and further research is still needed.

P53 is one of the senescence markers that exerts a role of anti-fibrosis by inhibiting cyclin protein, thus blocking the cell cycle and suppressing the proliferation of myofibroblasts^15,17,38^. We found that inhibition of TNAP induced upregulation of p53 in CFs through a TGF-β1/Smads-independent way. The process of p53-mediated senescence was further confirmed by analyzing apoptosis and cell cycle (Fig. 6e, f). Furthermore, p53 inhibitor PFT-α and genetic knockdown by si-P53 suppressed the protection effect of TNAP inhibition (Fig. 7 and Supplementary Fig. 6). Our results indicated that p53 signaling might be another potential mechanism for TNAP in regulating fibrosis. Nam et al.^39^ reported TNAP promotes calvarial progenitor cell cycle, which was consistent with what we have found in CFs. Therefore, inhibition of TNAP alleviated cardiac fibrosis probably through p53 signaling pathway. Further study is still needed to show how TNAP mediated p53 signaling and the cell circle of CFs.

In conclusion, TNAP could be a novel regulator in cardiac fibrosis and exert an antifibrotic effect mainly through AMPK-TGF-β1/Smads and p53 signals. However, as a tissue nonspecific protein, it is important to distinguish and identify the role of TNAP derived from different cells including CFs, myocytes, endothelial cells, and myeloid-origin cells such as macrophage. Although we explored the role of TNAP on the key fibrotic driving force of CFs, it keeps unclear whether TNAP mediated cardiac-resident CFs or myeloid-derived CFs, or both of them. Further research by using cell-specific TNAP transgenic mice is of great importance to fully understand the role of TNAP on cardiac fibrosis.

## Methods

### Study population

To investigate whether serum TNAP was upregulated after MI, patients from our previous cohort study^40^ were reviewed. It was approved by the Human Ethics Committee of the First Affiliated Hospital of Chongqing Medical University (Number 2016–39) with clinicaltrials.gov identifier NCT03462277. Another cohort (clinicaltrials.gov identifier NCT02812797) for prognostic study initially enrolled 1035 STEMI patients from a continuous sample in the same hospital from 17 September 2015 to 23 July 2017. Patients who were still alive from the start of our research were well-consented. Patients who had already met the end point were informed to their family members for consent. All the data were collected using the same protocol by well-trained researchers with a double-blind method. Both cohort studies were approved by the local ethic committee (Number 2018–035).

### Animals and human heart samples

Specific pathogen free (SPF) male SD rats (160–180 g, 6–8 W) were purchased from experimental animal center of Chongqing Medical University [certificate number SCXK (Yu) 2017–0001], raised in SPF animal experiment room (constant temperature 20 ± 3 °C with 55% ± 10% humidity, 12 h light/dark cycle). All animal experiments were performed according to institutional, local, and national guidelines on animal research and ethics. Human heart samples were from the death patients with MI. Patients or patients’ family members were well informed. The animals and human heart sections used for this study were approved by the local ethic committee (Number 2017–174).

### Reagents and antibodies

Tetra were from Sigma-Aldrich, Co. (T1512, St. Louis, MO, USA). Pifithrin-α hydrobromide was from MedChemExpress (HY-15484, Monmouth Junction, NJ, USA). Recombinant Human TGF-β1 Protein was from R&D Systems, Inc. (Minneapolis, MN, USA). The following primary antibodies were used: rabbit anti‐TNAP (ab108337, Abcam, Cambridge, UK), rabbit anti‐α-SMA (ab124964, Abcam), rabbit anti‐Vimentin (ab92547, Abcam), rabbit anti‐Fibronectin (ab2413, Abcam), rabbit anti‐TGF-β1 (SAB4502954, Sigma-Aldrich, Co.), rabbit anti-p-Smad2 (Ser465/Ser467) (#18338, Cell Signaling Technology, Danvers, MA, USA), rabbit anti-Smad2 (#5339, Cell Signaling Technology), rabbit anti-Smad3 (#9523, Cell Signaling Technology, Danvers, MA, USA), rabbit anti-AMPKα1/2 (phosphoThr183/172, YT0216, ImmunoWay Biotechnology, Co., Plano, TX, USA), rabbit anti-p-AMPKα1/2 (YP0575, ImmunoWay), rabbit anti-p53 (ab26, Abcam), rabbit anti-cyclinE (YT1176, ImmunoWay), and mouse anti-glyceraldehyde 3-phosphate dehydrogenase (GAPDH) (YM1038, ImmunoWay). Horseradish peroxidase (HRP)-conjugated goat anti-rabbit (RS0004) or anti-mouse (RS0001) were from ImmunoWay. Goat anti-rabbit IgG secondary antibodies Alexa Fluor® 488 (RS23220, ImmunoWay) were used for immunofluorescent analysis.

### Methods

#### Animal model of MI and TNAP inhibition

Rats were randomly divided into three groups (*n* = 8 per group): the normal control (Sham group), MI combined with saline injection (MI + Saline group), and MI combined with TNAP inhibitor, Tetra (MI + Tetra group). Tetra (11 mg/kg/day) and saline were administrated 7 days before MI. Rats were anesthetized with sodium pentobarbital (60 mg/kg, i.p.). The MI model was established by ligating the left anterior descending coronary artery. The control group was treated with a sham operation. All rats were sacrificed 14 days after MI by isoflurane (5%) administration and cervical dislocation.

#### Examination by echocardiography

Echocardiography were used to examine the cardiac function and ventricular structure by analyzing the parameters as follows: LV EF, LV FS, LV internal dimension (LVID), and interventricular septum thickness (IVS). The researcher was fixed and blinded.

#### Histological and IHC staining

Samples were fixed in 10% phosphate-buffered formalin and embedded in paraffin. Serial left ventricle cross-sections (6 μm thickness) were deparaffinized and stained with picric acid sirius red. The sections also incubated antibodies for TNAP (1:300), a-SMA (1:300), TGF-β1 (1:300), Fibronectin (1:300), and Vimentin (1:300), at 4 °C overnight. Other steps for IHC staining were according to the protocol of Two Step IHC Kits (PV-9000, ZDGB-BIO, Co., Beijing, China). Image analysis was performed by using the image pro plus 6.0 software.

#### Isolation and culture of CFs

Primary CFs were isolated from 1- to 3-day-old neonatal rats. Briefly, rats were anesthetized with isoflurane (5%) and sacrificed by cervical dislocation. Minced ventricles were digested with 0.04% collagenase II (Sigma-Aldrich, Co.) for six cycles. Cells were collected and suspended in Dulbecco’s modified Eagle medium: Nutrient Mixture F12 (DMEM/F12, Gibco, Thermo Fisher Scientific, Co., USA) containing 10 % fetal bovine serum (FBS, Wisent, Wisent Biomart, Co., Canada). After plating at 37 °C for 1 h, CFs attached to culture plates and the non-adherent cells in the supernatant were removed. CFs were cultured for 3–4 days until they reached confluence and were passaged further. The identity of CFs was confirmed by immunofluorescence for α-SMA. CFs at the third or fourth passages were used for experiments. After starvation in serum-free medium for 24 h, 10 ng/ml recombinant human TGF-β1 protein or hypoxia (1% O_2_) were applied to CFs in the presence or absence of 1 mM Tetra for 24 h and 48 h.

#### Cell migration assays

Migration of CFs was measured by transwell and wound-healing assays. For the transwell assay, CFs (1 × 10^4^cells/well) were added to the upper chamber of a 24-well cell culture chamber (8 μm pore size, Corning, NY, USA) in 100% serum-free DMEM/F12 with Tetra and/or TGF-β1 incubation. After 24 h, cells were fixed with 4% paraformaldehyde, stained with 0.5% methyl violet solution, and photographed. For wound-healing assay, CFs were grown to confluence in six-well plates and the bottom of monolayer cells was scraped off using a sterile p200 pipette tip. Cells were then treated with Tetra and/or TGF-β1 or hypoxia (1% O_2_) in serum-free or 10% serum DMEM/F12, and were allowed to migrate to the denuded area for 24 h and 48 h. In addition, the relative speed of migration was measured by the mean linear movement speed of wound edges.

#### Western blotting

Total protein was extracted in RIPA lysis buffer (Beyotime Biotechnology, Shanghai, China) supplemented with phenylmethylsulfonyl fluoride (PMSF, Beyotime) and protein phosphatase inhibitor (Beyotime). The protein concentrations were measured using the BCA Protein Assay (Beyotime). The samples were separated on a 10% SDS-polyacrylamide gel and then transferred to a nitrocellulose membrane, which was blocked in 5% skim milk for 1 h and then incubated with primary antibodies at 4 °C overnight. After the washing steps, the membranes were incubated with HRP-labeled secondary antibodies for 1 h at 37 °C. The bands were visualized using Immobilon Western HRP (Millipore, Germany). Relative band densities of proteins in western blottings were normalized against GAPDH.

#### TNAP activity assays

Alkaline phosphatase assay kit was from Nanjing Jiancheng Bioengineering Institute (A059-2-2, Nanjing, China). TNAP decomposes phenylene disodium phosphate to produce free phenol and phosphoric acid. Phenol reacts with 4-aminoantipyrine in alkaline solution and is oxidized by potassium ferricyanide to produce red quinone derivative. According to the depth of red, the activity of enzyme can be determined.

#### In-gel TNAP activity

SIGMAFAST™ BCIP^®^/NBT (B5655, Sigma-Aldrich) was used for in-gel TNAP activity assay. Total protein was extracted in RIPA lysis buffer without adding PMSF, protein phosphatase inhibitor, and SDS-polyacrylamide gel electrophoresis protein loading buffer. After measuring protein concentrations, the samples were separated on a 10% SDS-polyacrylamide gel. Gels were washed with BCIP/NBT solution according to the manufacturer’s instructions until clear bands appear. Gels were also stained with Coomassie blue (Beyotime) as references of total protein. TNAP hydrolyzes BCIP, generating NBT-formazan which is an insoluble blue–purple product and precipitates in gel with TNAP around 130 KD. The quantity of the precipitations indicates the activity of TNAP.

#### Quantitative real-time PCR

Total RNA of CFs was isolated by TRIzol reagent (Invitrogen) and 1 μg of total RNA was converted to cDNA using the QuantiTect Reverse Transcription Kit (Qiagen, Co., Germany). Quantitative real-time PCR was performed using QuantiNova SYBR Green PCR Kit (Qiagen, Co., Germany) and PCR primers were designed and synthesized by Sangon Biotech, Co. (Shanghai, China) as follows. All data were quantified by use of the comparative cycle threshold (CT) method.

**Table:** 

Gene name	Forward	Reverse
*Col1a1*	5′-CTGACTGGAAGAGCGGAGAG-3′	5′-GAGTGGGGAACACACAGGTC-3′
*Col1a2*	5′-GAGGGCAACAGCAGATTCA-3′	5′-GCGAGATGGCTTATTCGTTT-3′
*Col3a1*	5′-AGAATGGGGAGACTGGACCT-3′	5′-ATGCCTTGTAATCCTTGTGGA-3′
*Smad7*	5′-ACCACGAGTTCATGCAGCAG-3′	5′-AGATGACCTCCAGCCAGCAC-3′
*α-SMA*	5′-CAGGGAGTGATGGTTGGAAT-3′	5′-GGTGATGATGCCGTGTTCTA-3′
*P53*	5′-CCCAGGATGTTGCAGAGTTGTT-3′	5′-TTGAGAAGGGACGGAAGATGAC-3′

#### Immunofluorescent analysis

Paraffin sections was used for Smad3 immunofluorescent analysis. After dewaxing, antigen retrieval, and goat serum blocking, Smad3 antibody (1:100) were incubated at 4 °C overnight and then incubated with secondary antibodies Alexa Fluor® 488 at 37 °C for 2 h. Nuclei were stained with 4, 6-diamidino-2-phenylindole (Sigma-Aldrich). Fluorescent images were captured by Olympus Microscopy Fluorescence Imaging System (Olympus, China).

CFs isolated from neonatal rat heart were cultured on 96-well plates and fixed with 4% paraformaldehyde, permeabilized with 0.1% Triton X-100, and incubated with 1% bovine serum albumin in phosphate-buffered saline (PBS). Then, cells were incubated with antibodies for a-SMA at 4 °C overnight following the same steps above.

#### Flow cytometry analyses

CFs were starved with serum-free medium for 24 h and then incubated with Tetra in 10% FBS medium for another 24 h before collection. CFs were collected and resuspended in 500 μl PBS and flow cytometry was performed immediately using fluorescein isothiocyanate-conjugated Annexin V or allophycocyanin (APC) -conjugated Annexin V (CytoFLEX). CFs were isolated and fixed in 75% ethanol overnight at 4 °C, for the cell cycle analysis (CytoFLEX). CFs were then stained with DNA Prep. Different phases were determined based on DNA content.

#### Cell transfection

siRNAs targeting P53-coding sequence was designed and synthesized by Genepharma, Co. (Shanghai, China). Cell transfection with si-P53 was performed according to the protocol of Lipofectamine^TM^ 3000 Reagent (Invitrogen). Briefly, cells were seeded to be 70–90% confluent at transfection. Then the DNA–Lipofectamine 3000 complexes in serum-free medium were added to cells in culture medium. The siRNAs sequences are as follows:

**Table:** 

Target	Sequence
P53 (5′ to 3′)	CCACUCGAUGGAGAAUAUUTT AAUAUUCUCCAUCGAGUGGTT
Negative control (5′ to 3′)	UUCUCCGAACGUGUCACGUTT ACGUGACACGUUCGGAGAATT

### Statistical analysis

Normal distributed data were expressed as the mean ± SD. Skewed data were presented as medians with interquartile ranges. Comparisons among groups were performed by one-way analysis of variance and Mann–Whitney *U*-test according to the distribution of data. Dunn’s multiple comparison test was used to analyze differences when equal variances not assumed. The independent Student’s *T*-test and Mann–Whitney *U*-test were used for comparisons of two groups according to the distribution of data. The best TNAP cutoff was that of the highest product of sensitivity and specificity for in-hospital mortality prediction. Cumulative incidence curves of in-hospital mortality were estimated using the Kaplan–Meier product–limit estimation method with the log-rank test. Cox proportional hazards model was used to analyze the independent effect of TNAP on in-hospital and follow-up mortality. All data analyses were performed by SPSS 22.0 statistical software. Statistical significance was defined as *P* < 0.05.

## Supplementary information

Supplemental table 1

Supplemental table 2

Supplemental table 3

Supplemental table 4

Supplemental figure legands

Supplemental figure 1

Supplemental figure 2

Supplemental figure 3

Supplemental figure 4

Supplemental figure 5

Supplemental figure 6

AUTHOR CONTRIBUTIONS

Reporting Checklist

Approval for TNAP

Approval for prognostic

Approval for coronary AS
