# Supplementary material for: TNAP inhibition attenuates cardiac fibrosis induced by myocardial infarction through deactivating TGF-β1/Smads and activating P53 signaling pathways
Source: Cell Death Dis. 2020 Jan 22;11(1):44. doi: 10.1038/s41419-020-2243-4 (PMC6976710; doi:10.1038/s41419-020-2243-4)
Supplement: Supplementary file 1 — Supplemental table 1 [file 41419_2020_2243_MOESM1_ESM.docx]

Supplemental Table 1. Baseline characteristics of ACS patients categorized by UA and AMI.

| **Characteristics*** | **Total (*n*=56)** | **UA (*n*=29)** | | **AMI (*n*=27)** | ***P* value** |
| --- | --- | --- | --- | --- | --- |
| Age, y | 75 (69, 78) | | 74 (68, 78) | 75 (70, 77) | 0.895 |
| Male (%) | 37 (66.1) | | 22 (75.9) | 15 (55.6) | 0.109 |
| TNAP | 70 (57, 88) | | 61 (57, 69) | 86 (72, 102) | 0.001 |
| T2DM (%) | 25 (44.6) | | 12 (41.4) | 13 (48.1) | 0.611 |
| Primary hypertension (%) | 40 (71.4) | | 23 (79.3) | 17 (63.0) | 0.259 |
| Gensini Score | 61.5 (29.0, 90.5) | | 47.0 (23.0, 86.5) | 72.0 (42.0, 90.5) | 0.216 |

T2DM, type 2 diabetes mellitus. Acute coronary syndrome, ACS. Unstable angina, UA. Acute myocardial infarction, AMI.

*Continuous variables were all skewed distribution and expressed as median (interquartile range). Categorical variables were expressed as frequency (percent).
