# Supplementary material for: TNAP inhibition attenuates cardiac fibrosis induced by myocardial infarction through deactivating TGF-β1/Smads and activating P53 signaling pathways
Source: Cell Death Dis. 2020 Jan 22;11(1):44. doi: 10.1038/s41419-020-2243-4 (PMC6976710; doi:10.1038/s41419-020-2243-4)
Supplement: Supplementary file 2 — Supplemental table 2 [file 41419_2020_2243_MOESM2_ESM.docx]

Supplemental Table 2. Baseline characteristics of study participants by Serum TNAP concentration, U/L.

| Characteristics* | Total (*n*=826) | <109 (*n*=624) | ≥109 (*n*=202) | *P* value |
| --- | --- | --- | --- | --- |
| n | 826 | 624 | 202 |  |
| Demographics |  |  |  |  |
| Age, y | 64 (54-73) | 66 (57-74) | 62 (56-67) | 0.325 |
| Male (%) | 620 (75.1) | 495 (79.3) | 125 (61.9) | <0.001 |
| Clinical features |  |  |  |  |
| sBP, mmHg | 123(107-142) | 123 (108-145) | 132 (116-140) | 0.194 |
| dBP, mmHg | 74 (65-89) | 72 (66-83) | 86 (73-92) | 0.021 |
| Admission heart rate, /min | 80 (68-92). | 79 (69-91) | 85 (71-96) | 0.015 |
| Triglyceride, mmol/L | 1.43 (0.96-2.145) | 1.43 (1.01-2.04) | 2.03 (1.23-2.04) | 0.023 |
| Total cholesterol, mmol/L | 4.26 (3.54-5.00) | 4.15 (3.45-4.71) | 4.97 (4.49-5.97) | 0.006 |
| HDL-C, mmol/L | 1.09 (0.90-1.30) | 1.06 (0.89-1.25) | 1.16 (0.75-1.60) | 0.731 |
| LDL-C, mmol/L | 2.74 (2.13-3.42) | 2.64 (2.13-3.14) | 3.29 (2.83-4.67) | 0.051 |
| apoA1, g/L | 1.17 (1.02-1.32) | 1.14 (1.03-1.29) | 1.25 (0.96-1.52) | 0.169 |
| apoB, g/L | 0.92 (0.75-1.11) | 0.88 (0.75-1.08) | 1.11 (0.98-1.21) | 0.037 |
| Lp (a), g/L | 115 (54-316) | 100 (50-294) | 258 (85-336) | 0.539 |
| hsCRP, mg/L | 7.16 (2.86-20. 00) | 6.20 (2.37-15.21) | 10.62 (7.93-20.00) | 0.001 |
| WBC, 10^9^ | 10.30 (8.34-12.77) | 10.03 (8.29-12.59) | 10.76 (8.78-13.08) | 0.005 |
| Cardiac troponin T, μg/L | 2.68 (0.32-14.00) | 1.53 (0.20-6.17) | 0.90 (0.39-1.48) | 0.561 |
| CK-MB, μg/L | 16.9 (3.0-62.0) | 4.7 (1.7-28.0) | 1.4 (1.0-1.9) | 0.303 |
| Urea, mmol/L | 5.9 (4.6-7.1) | 6.0 (5.2-7.1) | 4.9 (4.0-6.0) | 0.828 |
| Creatinine, μmol/L | 75 (64-93) | 75 (67-95) | 68 (62-92) | 0.967 |
| Sodium, mmol/L | 141 (139-143) | 141 (139-143) | 142 (141-145) | 0.573 |
| Potassium, mmol/L | 4.0 (3.7-4.3) | 4.1 (3.9-4.3) | 4.3 (3.9-4.5) | 0.698 |
| Calcium, mmol/L | 2.23 (2.13-2.31) | 2.23 (2.15-2.33) | 2.22 (2.13-2.36) | 0.188 |
| Total protein, g/L | 65 (61-70) | 64 (61-68) | 67 (61-70) | <0.001 |
| Albumin, g/L | 39 (35-42) | 38 (36-41) | 39 (37-43) | 0.013 |
| ALT, U/L | 40 (29-59) | 31 (20-53) | 32 (20-51) | <0.001 |
| AST, U/L | 98 (44-217) | 52 (22-120) | 39 (35-63) | 0.083 |
| TNAP, U/L | 86 (70-106) | 72 (63-88) | 127 (120-140) | <0.001 |
| Echocardiography |  |  |  |  |
| LVEF, % | 57 (52-60) | 57 (53-61) | 54 (51-56) | 0.044 |
| LVFS, % | 30 (27-32) | 30 (27-33) | 28 (27-29) | 0.014 |
| Medical history |  |  |  |  |
| Primary hypertension (%) | 424 (51.3) | 315 (50.5) | 109 (54.0) | 0.390 |
| T2DM (%) | 183 (22.2) | 133 (21.3) | 50 (24.8) | 0.306 |
| CKD (%) | 14 (1.7) | 8 (1.3) | 6 (3.0) | 0.192 |
| Stroke (%) | 29 (3.5) | 22 (3.5) | 7 (3.5) | 0.968 |
| Peripheral atherosclerosis (%) | 5 (0.6) | 5 (0.8) | 0 (0.0) | 0.342 |
| Peripheral arterial diseases (%) | 10 (1.2) | 8 (1.3) | 2 (1.0) | 1.000 |
| Therapy |  |  |  |  |
| β-receptor blocker (%) | 701 (84.9) | 527 (84.5) | 174 (86.1) | 0.562 |
| ACEI/ARB (%) | 614 (74.3) | 461 (73.9) | 153 (75.7) | 0.598 |
| Statin therapy (%) | 669 (81.0) | 505 (80.9) | 164 (81.2) | 0.935 |
| PCI | 676 (81.8) | 516 (82.7) | 160 (79.2) | 0.264 |
| Antiplatelet therapy | 826 (100.0) | 624 (100.0) | 202 (100.0) | - |
| Abbreviations: sBP, systolic blood pressure; dBP, diastolic blood pressure; HDL-C, high-density lipoprotein-cholesterol; LDL-C, low-density lipoprotein-cholesterol; apoA1, apolipoprotein AI; apoB, apolipoprotein B; Lp(a), lipoprotein(a); hsCRP, high sensitivity C reactive protein; FPG, fasting plasma glucose; CK-MB, Creatine kinase isoenzyme-MB; ALT, alanine aminotransferase; AST, Aspartate aminotransferase; LVEF, left ventricular ejection fraction; LVFS, left ventricular shortening fraction; T2DM, type 2 diabetes mellitus; CKD, chronic kidney disease; PCI, percutaneous coronary intervention.  *Continuous variables were all skewed distribution and expressed as median (interquartile range). Categorical variables were expressed as frequency (percent). | | | | |
