# Supplementary material for: TNAP inhibition attenuates cardiac fibrosis induced by myocardial infarction through deactivating TGF-β1/Smads and activating P53 signaling pathways
Source: Cell Death Dis. 2020 Jan 22;11(1):44. doi: 10.1038/s41419-020-2243-4 (PMC6976710; doi:10.1038/s41419-020-2243-4)
Supplement: Supplementary file 3 — Supplemental table 3 [file 41419_2020_2243_MOESM3_ESM.docx]

Supplemental Table 3. Results of univariate Cox proportional hazards model applied to assess correlates of in-hospital mortality.

| **Variables*** | **In-hospital Mortality** | |
| --- | --- | --- |
|  | HR [95% CI] | *P* value |
| TNAP (≥109) | 4.180 [1.450, 12.048] | 0.008 |
| Age, per 10 year | 1.009 [1.004, 1.015] | 0.001 |
| Male | 0.090 [0.025, 0.322] | <0.001 |
| sBP, per mmHg | 0.988 [0.967, 1.009] | 0.258 |
| dBP, per mmHg | 0.956 [0.929, 0.984] | 0.002 |
| Admission heart rate, per 10 | 1.001 [1.001, 1.002] | <0.001 |
| LVEF, per 1% | 1.038 [0.907, 1.188] | 0.592 |
| Sodium, per mmol/L | 0.971 [0.854, 1.103] | 0.649 |
| Potassium, per mmol/L | 2.371 [1.117, 5.034] | 0.025 |
| Calcium, per mmol/L | 0.254 [0.035, 1.853] | 0.176 |
| Total cholesterol, per mmol/L | 1.230 [0.789, 1.917] | 0.360 |
| Triglyceride, per mmol/L | 0.780 [0.408, 1.492] | 0.454 |
| HDL-C, per mmol/L | 1.508 [0.869, 2.619] | 0.144 |
| LDL-C, per mmol/L | 1.114 [0.676, 1.837] | 0.672 |
| apoA1, per g/L | 1.517 [0.784, 2.937] | 0.216 |
| apoB, per g/L | 1.068 [0.169, 6.744] | 0.944 |
| Lp (α), per g/L | 1.000 [0.998, 1.002] | 0.797 |
| hsCRP, per mg/L | 1.064 [0.985, 1.149] | 0.116 |
| WBC, per 10^9^ | 1.236 [1.119, 1.365] | <0.001 |
| Cardiac troponin T, per μg/L | 1.000 [0.989, 1.012] | 0.947 |
| CK, MB, per μg/L | 1.002 [0.998, 1.007] | 0.360 |
| Creatinine, per μmol/L | 1.004 [1.002, 1.006] | <0.001 |
| Urea, per mmol/L | 1.082 [1.020, 1.147] | 0.008 |
| Total protein, per g/L | 1.017 [0.965, 1.072] | 0.525 |
| Albumin, per g/L | 0.963 [0.896, 1.035] | 0.308 |
| β receptor blocker therapy | 0.398 [0.125, 1.272] | 0.120 |
| ACEI/ARB therapy | 0.087 [0.024, 0.311] | <0.001 |
| PCI therapy | 0.256 [0.089, 0.740] | 0.012 |
| Primary hypertension history | 2.423 [0.760, 7.726] | 0.135 |
| T2DM history | 1.949 [0.653, 5.815] | 0.232 |
| CKD history | 9.923 [2.220, 44.349] | 0.003 |
| Stroke history | 2.334 [0.305, 17.851] | 0.414 |

*TNAP was categorized as ≥109 U/L and lower than that.

HRs were increased hazard ratios of per 1 or per 10 unit increasing of variables.

HRs of categorical variables were hazard ratios of yes to no.
