# Supplementary material for: TNAP inhibition attenuates cardiac fibrosis induced by myocardial infarction through deactivating TGF-β1/Smads and activating P53 signaling pathways
Source: Cell Death Dis. 2020 Jan 22;11(1):44. doi: 10.1038/s41419-020-2243-4 (PMC6976710; doi:10.1038/s41419-020-2243-4)
Supplement: Supplementary file 4 — Supplemental table 4 [file 41419_2020_2243_MOESM4_ESM.docx]

Supplemental Table 4. Results of multivariate Cox proportional hazards model applied to assess correlates of in-hospital mortality.

| **Variables*** | **Mode 1** | | **Mode 2** | | **Mode 3** | |
| --- | --- | --- | --- | --- | --- | --- |
|  | HR [95% CI] | *P* value | HR [95% CI] | *P* value | HR [95% CI] | *P* value |
| TNAP (≥109) | 3.158 [1.074, 9.289] | 0.037 | 4.455 [1.309, 15.161] | 0.017 | 4.595 [1.079, 19.574] | 0.039 |
| Age, per 10 year | 1.007 [1.002, 1.013] | 0.005 | 1.007 [1.001, 1.013] | 0.014 | 1.012 [1.004, 1.019] | 0.004 |
| Male | 0.182 [0.048, 0.693] | 0.012 | 0.311 [0.075, 1.294] | 0.108 | 0.160 [0.020, 1.300] | 0.087 |
| dBP, per mmHg |  |  | 0.959 [0.929, 0.990] | 0.009 | 0.956 [0.917, 0.997] | 0.035 |
| Admission heart rate, per 10 |  |  | 1.001 [1.001, 1.002] | <0.001 | 1.001 [1.000, 1.002] | 0.044 |
| Potassium, per mmol/L |  |  |  |  | 2.199[0.788, 6.132] | 0.132 |
| WBC, per 10^9^ |  |  |  |  | 1.369 [1.154, 1.623] | <0.001 |
| Creatinine, per μmol/L |  |  |  |  | 1.005 [0.999, 1.011] | 0.126 |
| ACEI/ARB therapy |  |  |  |  | 0.224 [0.038, 1.310] | 0.097 |
| PCI therapy |  |  |  |  | 0.922 [0.159, 5.332] | 0.928 |
| CKD history |  |  |  |  | 2.653 [0.093, 75,778] | 0.568 |

Mode 1: adjusted for age and male.

Mode 2: adjusted for Mode 1 and further adjusted for admission diastolic BP, and admission heart rate.

Mode 3: adjusted for Mode 2 and further adjusted for potassium, WBC, Creatinine, ACEI/ARB therapy, PCI therapy and CKD history.

*TNAP was categorized as ≥109 U/L and lower than that.

HRs were increased hazard ratios of per 1 or per 10 unit increasing of variables.

HRs of categorical variables were hazard ratios of yes to no.
