# Supplementary material for: TNAP inhibition attenuates cardiac fibrosis induced by myocardial infarction through deactivating TGF-β1/Smads and activating P53 signaling pathways
Source: Cell Death Dis. 2020 Jan 22;11(1):44. doi: 10.1038/s41419-020-2243-4 (PMC6976710; doi:10.1038/s41419-020-2243-4)
Supplement: Supplementary file 5 — Supplemental figure legands [file 41419_2020_2243_MOESM5_ESM.docx]

**Supplementary Figures for**

**TNAP inhibition attenuates cardiac fibrosis induced by myocardial infarction through deactivating TGF-β1/Smads and activating P53 signaling pathways**

Lei GAO^1^, Li-you WANG^1^, Zhi-qiang LIU^1^, Dan JIANG^1^, Shi-yong WU^1^, Yu-qian GUO^1^, Hong-mei TAO^1^, Min Sun^1^, Lin-na YOU^1^, Shu QIN^1^, Xiao-cheng CHENG^1^, Jun-shi XIE^1^, Guang-lei CHANG^1^*, Dong-ying Zhang^1^*

^1^Department of Cardiovascular Medicine, The First Affiliated Hospital of Chongqing Medical University, Chongqing 400016, China

**Supplemental figure 1. Trial flowchart of figure 1B-D.**

**Supplemental figure 2. TNAP activity and expression was increased after MI injury by a time dependent manner in SD rats.**

A. Time axis of SD rats. Rats were suffered MI or sham operation at 28 days, 14 days, 7 days, 3 days and 1 day before sacrificing. B. α-SMA, TANP and GAPDH measured by western blotting. (n=5 for each group). C. Collagen deposition and TNAP expression measured by sirus red staining and IHC. (n=4 for each group). Bar, 50 μm. D. Serum TNAP activity assay by alkaline phosphatase assay kit. E. Heart TNAP activity assay by alkaline phosphatase assay kit. F. In-gel heart TNAP activity assay by BCIP/NBT method. TNAP was around 130 KD.

**Supplemental figure 3. Tetra administration continuously inhibited heart TNAP activity in rats.**

A. Time axis of SD rats. Rats were injected with Tetra (11mg/kg/day) or saline (same volume with Tetra solution) once a day each time point before sacrificing. B. Serum TNAP activity measured by alkaline phosphatase assay kit. (n=3 for sham group, n=4 for other four groups). C. Heart TNAP activity by alkaline phosphatase assay kit. (n=3 for sham group, n=4 for other four groups). D. In-gel heart TNAP activity by BCIP/NBT method. (n=3 for each group). TNAP was around 130 KD.

**Supplemental figure 4. Activation of AMPK and deactivation of Smad2/3 signaling was involved in Tetra administration in rats after MI.**

A. p-AMPK, AMPK and GAPDH expression (n=3, each). B. pSmad2, Smad2 and GAPDH expression (n=3, each). C. Smad3 expression. (n=3, each). Arrows shows the transportation to the nucleus.

**Supplemental figure 5. Inhibition TNAP improved hypoxia-induced morphology changes of CFs.** (n=3, each).

**Supplemental figure 6. The anti-fibrotic effect of inhibition TNAP was probably through p53 signaling.**

A. P53 and p21 measured by western blotting after transfection for 72h. Cells were transfected by si-P53-Lipofectamine 3000 complexes in serum-free medium for 24h. Then the medium was changed to 10% FBS DMEM/F12 and cultured for another 48h before harvest. B. P53 mRNA expression measured by RT-PCR. After transfection for 24h in serum-free medium, cells were treated with Tetra and incubated in hypoxia (1% O_2_) for another 24h before harvest. C. Migration was assessed by transwell and woundhealing assays. For transwell, cells were transfected for 24h, resuspending in serum-free medium and seeding in wells for 24h before photographing. For woundhealing, after transfection for 24h, the medium was changed to 10% FBS with the presence or absence of Tetra and incubated in normoxia or hypoxia for another 24h before photographing. (n=3 for each experiment). NC groups were transfected with negative control siRNA. ***P*<0.01 *vs.* NC group. #*P*<0.05 *vs*. Hypoxia+NC group. ^&&^*P*<0,01 *vs.* Normoxia+NC+Tetra group. ^$$^*P*<0.01 *vs.* Hypoxia+NC+Tetra group.
