# Supplementary figures and images for: TNAP inhibition attenuates cardiac fibrosis induced by myocardial infarction through deactivating TGF-β1/Smads and activating P53 signaling pathways
Source: Cell Death Dis. 2020 Jan 22;11(1):44. doi: 10.1038/s41419-020-2243-4 (PMC6976710; doi:10.1038/s41419-020-2243-4)

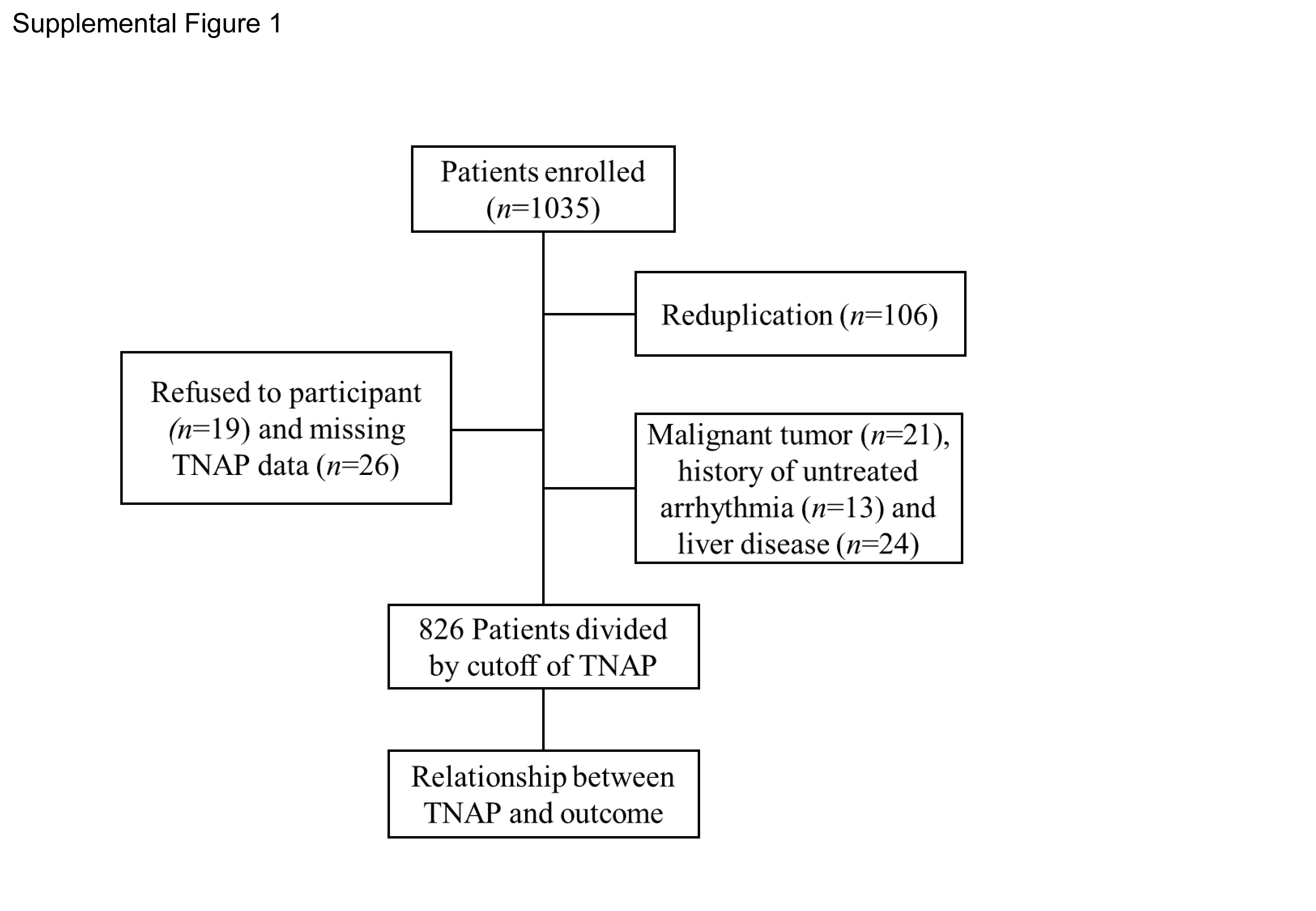

Supplement: Supplementary file 6 — Supplemental figure 1 [file 41419_2020_2243_MOESM6_ESM.tif]

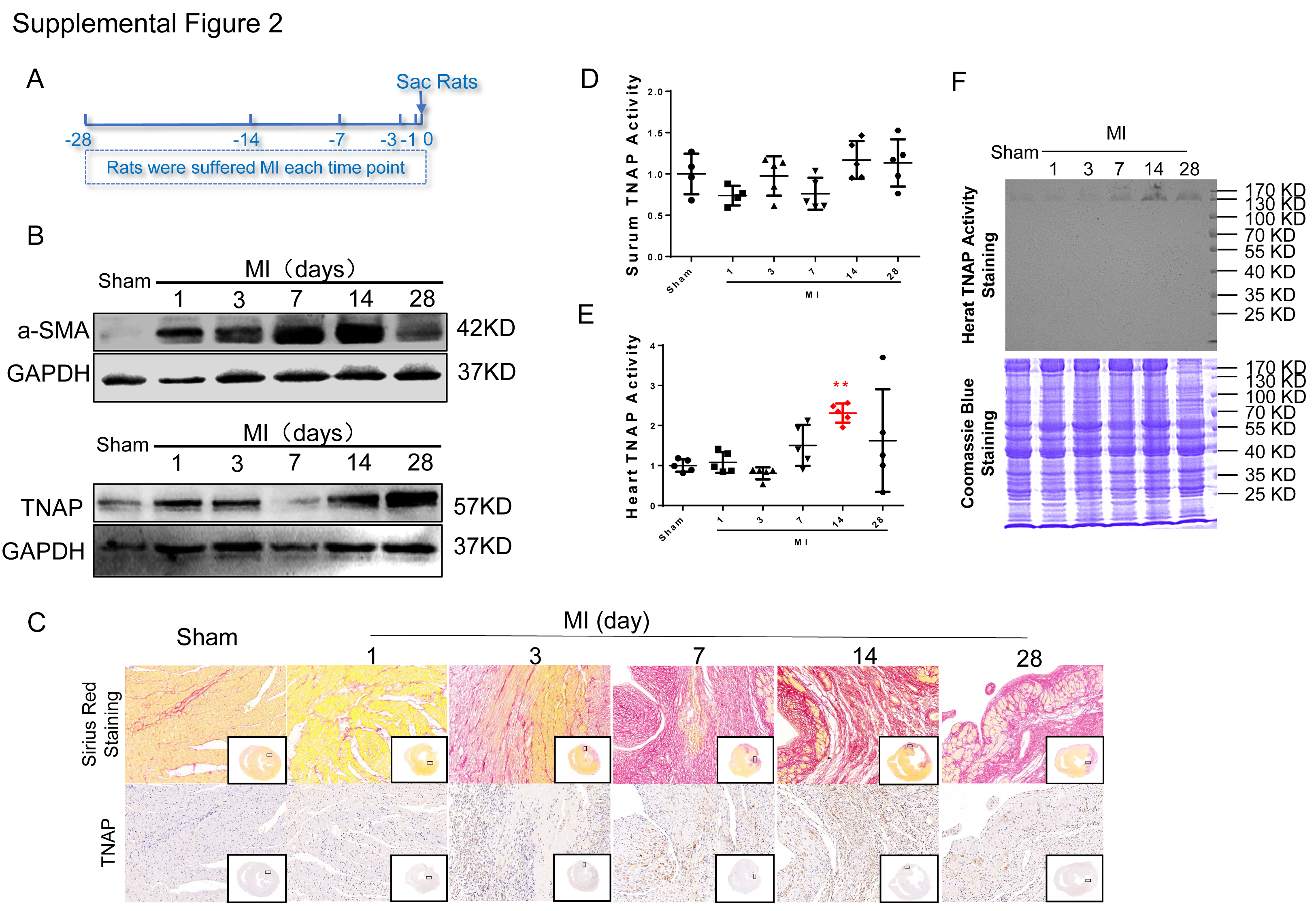

Supplement: Supplementary file 7 — Supplemental figure 2 [file 41419_2020_2243_MOESM7_ESM.tif]

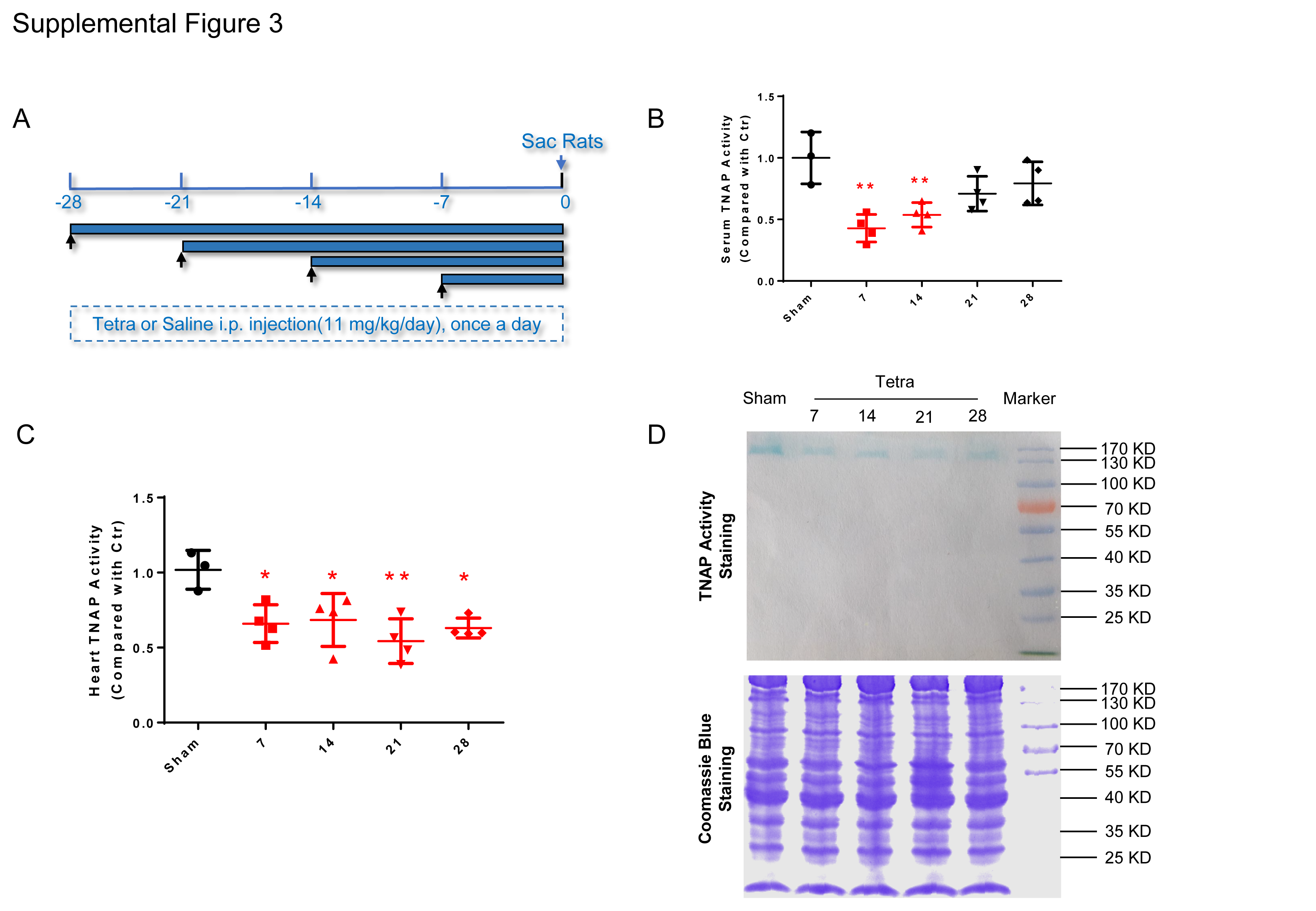

Supplement: Supplementary file 8 — Supplemental figure 3 [file 41419_2020_2243_MOESM8_ESM.tif]

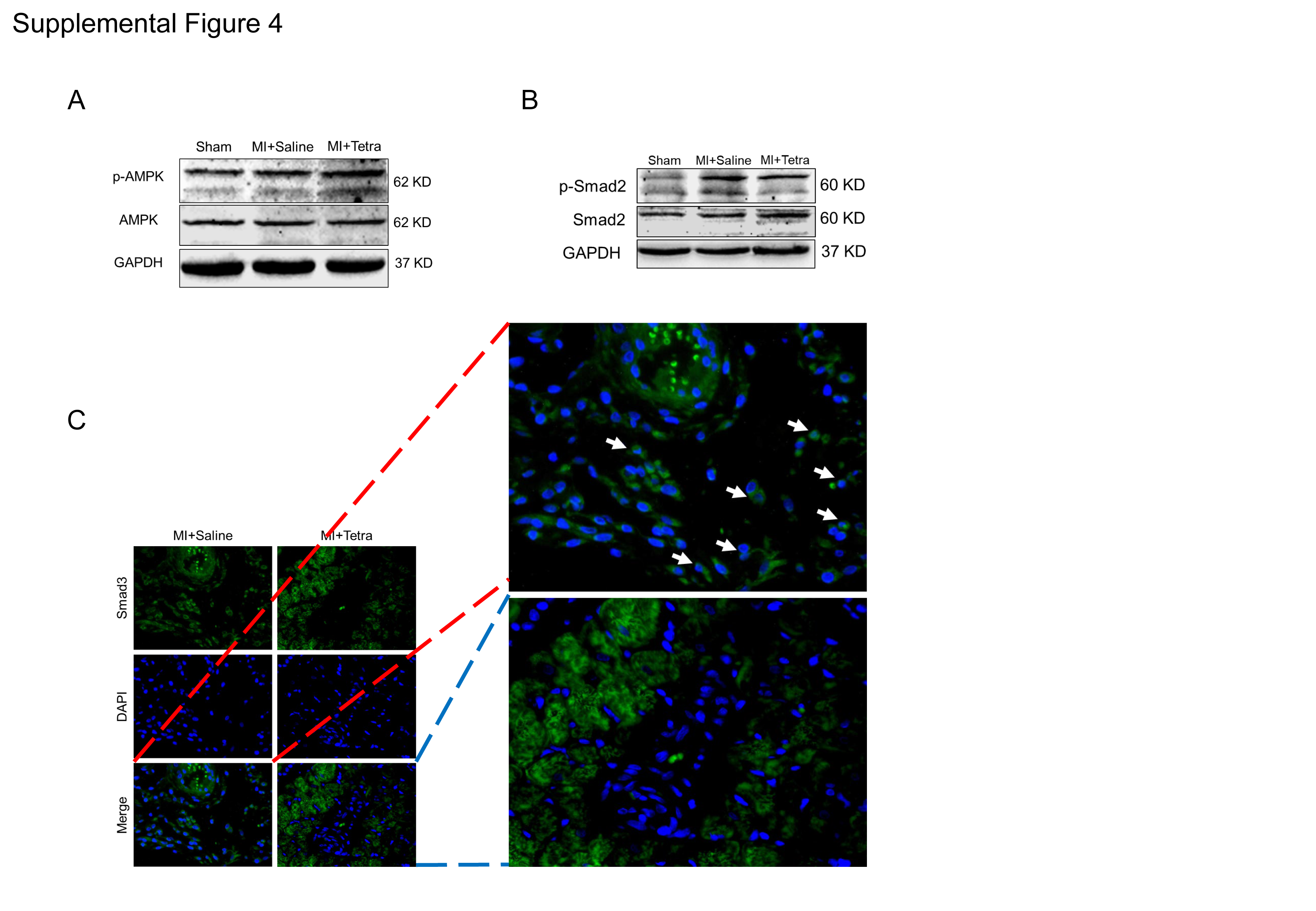

Supplement: Supplementary file 9 — Supplemental figure 4 [file 41419_2020_2243_MOESM9_ESM.tif]

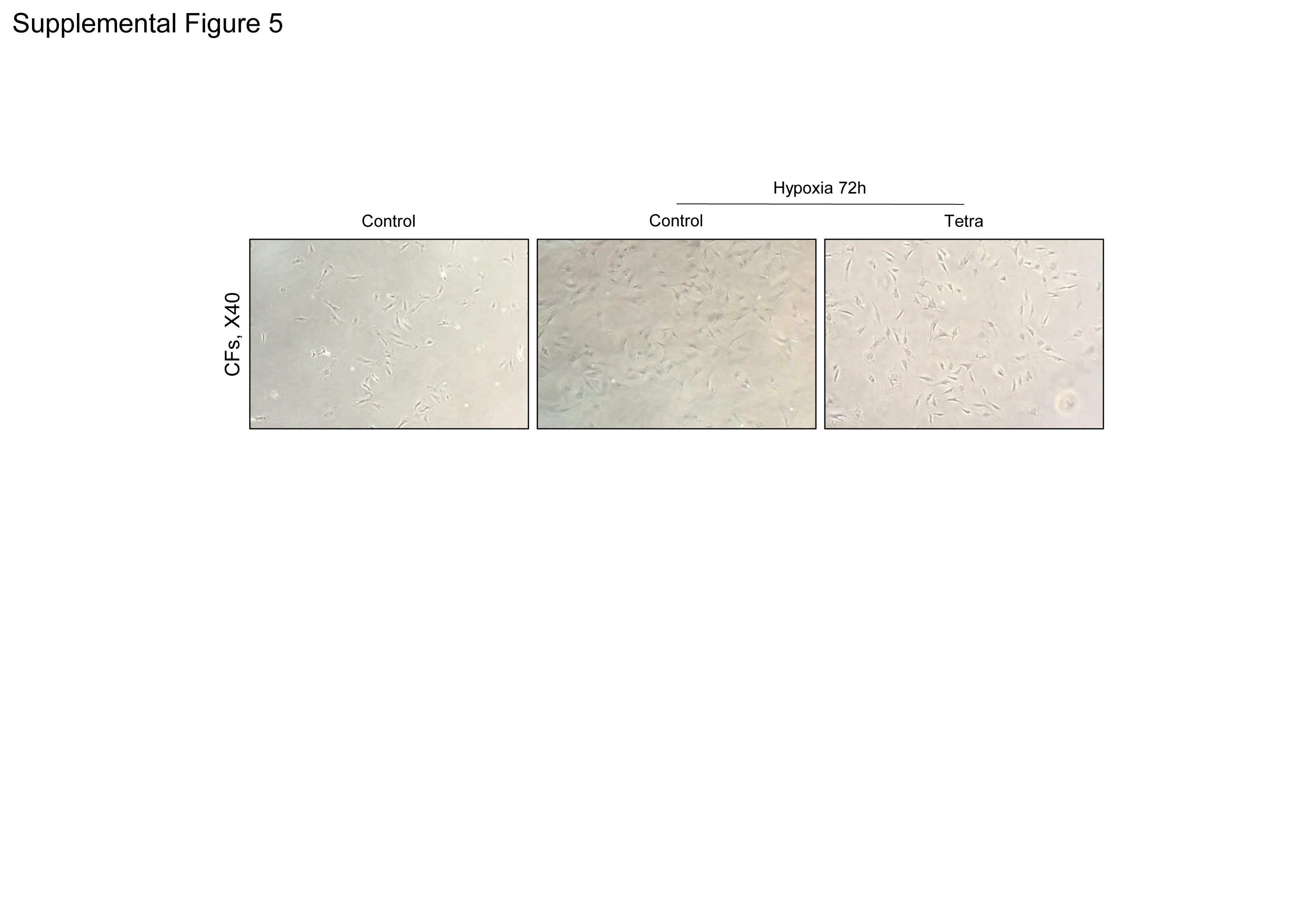

Supplement: Supplementary file 10 — Supplemental figure 5 [file 41419_2020_2243_MOESM10_ESM.tif]

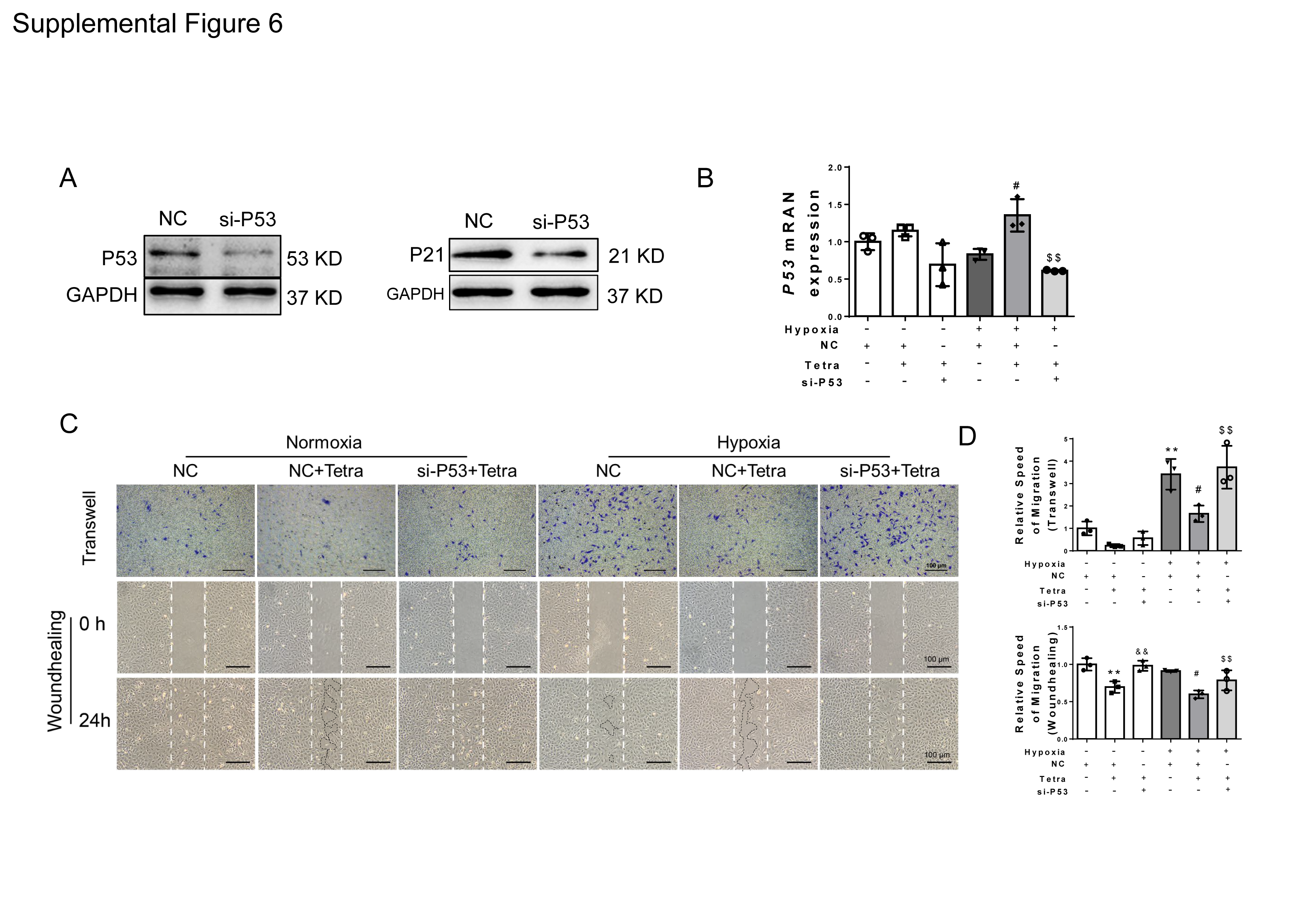

Supplement: Supplementary file 11 — Supplemental figure 6 [file 41419_2020_2243_MOESM11_ESM.tif]
