# Supplementary material for: TNAP inhibition attenuates cardiac fibrosis induced by myocardial infarction through deactivating TGF-β1/Smads and activating P53 signaling pathways
Source: Cell Death Dis. 2020 Jan 22;11(1):44. doi: 10.1038/s41419-020-2243-4 (PMC6976710; doi:10.1038/s41419-020-2243-4)
Supplement: Supplementary file 12 — AUTHOR CONTRIBUTIONS [file 41419_2020_2243_MOESM12_ESM.pdf]

DECLARATION OF CONTRIBUTIONS TO ARTICLE

ADMC

Manuscript Number: CDDIS-19-3481R

Journal Name: *Cell Death & Disease* (the 'Journal')

Proposed Title of the Contribution:

TNAP Inhibition Attenuates Cardiac Fibrosis Induced by Myocardial Infarction through Deactivating TGF-β1/Smads and Activating P53 Signaling Pathways (the 'Contribution')

Author(s):

Lei GAO, Li-you WANG, Zhi-qiang LIU, Dan JIANG, Shi-yong WU, Yu-qian GUO, Hong-mei TAO, Min Sun, Lin-na YOU, Shu QIN, Xiao-cheng CHENG, Jun-shi XIE, Guang-lei CHANG, Dong-ying Zhang (the 'Authors')

For all *CDDis* articles, each person named as an author in the published version must be able to show he or she has contributed substantially to the article.

Authorship credit should be based on 1) substantial contributions to conception and design, acquisition of data, or analysis and interpretation of data; 2) drafting the article or revising it critically for important intellectual content; and 3) final approval of the version to be published. Authors should meet conditions 1, 2 and 3.

Any person who cannot be shown to have made a substantial contribution to the article cannot be listed as an author in the final version. The name of any person who is deemed to have made a minor contribution can, however, appear in the Acknowledgments section of the article.

Please complete the table below to indicate the contributions of all named authors to the manuscript.

Author Full Name:	Specification of Contribution to the Manuscript:
Lei GAO	Design, acquisition of data, analysis data; drafting the article and revising it.
Li-you WANG	Experiments and data collection
Zhi-qiang LIU	Experiments and data collection
Dan JIANG	Clinical data collection of AMI patients
Shi-yong WU	Experiments and data collection
Yu-qian GUO	Experiments and data collection
Hong-mei TAO	Clinical experiment design and data collection
Min Sun,Lin-na YOU	Following-up patients, clinical data collection
Shu QIN	Experimental design, supervision
Xiao-cheng CHENG	Experiments and data collection
Jun-shi XIE	Clinical data collection
Guang-lei CHANG	Design, drafting the article and revising it
Dong-ying Zhang	Design, drafting the article and revising it, final approval of the version to be published

Please complete the table below to indicate the contributions of all named authors to the figures.

Figure 1:

Contributed author list:Lei GAO, Hong-mei TAO, Dan Jiang, Li-you Wang, Min Sun, Lin-na YOU, Jun-shi Xie, Shu Qin, Dong-ying ZHANG

Details: Dan Jiang contributed to the collection of data used in Figure1A. Hong-mei TAO and Jun-shi Xie contributed to collection the clinical data in Figure1B-D. Min Sun and Lin-na You contributed to the following-up of patients. Liyou Wang contributed to human heart sample collection. Xiaocheng Cheng contributed to drafting figure1D and analyzing clinical data. Lei Gao was involved in all these work mentioned above. Shu Qin and Dong-ying Zhang contributed to work supervision and design.

Figure 2:

Contributed author list: Lei GAO, Shi-yong Wu,Guang-lei CHANG, Dong-ying ZHANG

Details: Shi-yong Wu contributed to animal model setting up. Lei Gao contributed to carrying out the research work, analyzing data and drafting figures. Guanglei Chang and Dong-ying Zhang contributed to work supervision and design.

Figure 3:

Contributed author list: Lei GAO, Zhi-qiang LIU, Li-you WANG, Shi-yong WU, Guang-lei CHANG, Dong-ying ZHANG

Details: Li-you Wang and Shi-yong Wu were involved in the work of echocardiography. Zhi-qiang Liu contributed to the the experiments of heart TNAP activity assays. Lei Gao was involved in all the work in this figure. Guanglei Chang and Dong-ying Zhang contributed to work supervision, direction and design.

Figure 4:

Contributed author list:Lei GAO, Li-you WANG,Zhi-qiang LIU, ,Guang-lei CHANG, Dong-ying ZHANG

Details: Li-you Wang helped to performing IHC staining. Zhiqiang LIU helped to running western blotting. Lei Gao was involved in all the work in this figure. Guanglei Chang and Dong-ying Zhang contributed to work supervision, direction and design.

Figure 5:

Contributed author list: Lei GAO, Yu-qian GUO,Guang-lei CHANG, Dong-ying ZHANG

Details: Yu-qian GUO helped to cell culturing. Lei Gao was involved in all the work in this figure. Guanglei Chang and Dong-ying Zhang contributed to work supervision, direction and design.

Figure 6:

Contributed author list: Lei GAO, Zhi-qiang LIU, Yu-qian GUO, Guang-lei CHANG, Dong-ying ZHANG

Details: Yu-qian GUO and Zhi-qiang Liu helped to cell culturing. Zhi-qiang Liu also contributed to flow cytometry. Lei Gao was involved in all the work in this figure. Guanglei Chang and Dong-ying Zhang contributed to work supervision, direction and design.

Special notes: Zhi-qiang Liu and Yu-qian Guo also contributed the work of figure 7.

Signed for and on behalf of the Author(s):	Print Name:	Date:
Dong-ying Zhang	Dong-ying ZHANG	2019.12.20
