# Supplementary material for: TNAP inhibition attenuates cardiac fibrosis induced by myocardial infarction through deactivating TGF-β1/Smads and activating P53 signaling pathways
Source: Cell Death Dis. 2020 Jan 22;11(1):44. doi: 10.1038/s41419-020-2243-4 (PMC6976710; doi:10.1038/s41419-020-2243-4)
Supplement: Supplementary file 14 — Approval for TNAP [file 41419_2020_2243_MOESM14_ESM.pdf]

The Ethics Committee

The First Affiliated Hospital of ChongQing Medical University

Approval Notice

Principal Investigators: Dong-ying Zhang

Title of Projects: Effect of TNAP on Cardiac Fibrosis.

Date Reviewed: 2017-12-11

Date Approved: 2017-12-11

Approval number: 2017-174

The Ethics Committee of The First Affiliated Hospital has reviewed the proposed use of human and animal subjects in the above mentioned projects. It is recognized that the rights and the welfare of the subjects are adequately protected; the potential risks are outweighed by potential benefits.

The Ethics Committee
The First Affiliated Hospital of ChongQing Medical University

2017-12-11
